# Improved detection of homologous recombination deficiency in Chinese patients with ovarian cancer: a novel non‐exonic single‐nucleotide polymorphism‐based next‐generation sequencing panel

**DOI:** 10.1002/1878-0261.13411

**Published:** 2023-03-16

**Authors:** Bing Wei, Jinxiang Zheng, Cai Jiang, He Zhang, Mingye Zhang, Taoran Cheng, Jun Li, Zhizhong Wang, Lijun Deng, Li Wang, Qingxin Xia, Jie Ma

**Affiliations:** ^1^ The Affiliated Cancer Hospital of Zhengzhou University & Henan Cancer Hospital China; ^2^ Department of Molecular Pathology Henan Key Laboratory of Molecular Pathology Zhengzhou China; ^3^ Nanodigmbio (Nanjing) Biotechnology Co., Ltd. China

**Keywords:** Chinese population, CNV, HRD, ovarian cancer, SNP‐based NGS panel

## Abstract

As homologous recombination deficiency (HRD) is a biomarker to predict the efficiency of PARP inhibitor treatment, this study developed a non‐exonic single‐nucleotide polymorphism (SNP)‐based targeted next‐generation sequencing panel and comprehensively examined it both on standard and clinical ovarian cancer tissues. The HRD scores calculated by the panel and whole‐genome sequencing were consistent, with the analysis by sequenza being the most reliable. The results on clinical samples revealed that the panel performed better in HRD analysis compared with the SNP microarray. There are several distinctions between this newly developed kit and reported HRD detection panels. First, the panel covers only 52 592 SNPs, which makes it capable of detecting genomic instability. Secondly, all the SNPs are non‐exonic; as a result, the panel can be used cooperatively with any exon panel. Thirdly, all the SNPs selected have a high minor allele frequency in Chinese people, making it a better choice for HRD detection in Chinese patients. In summary, this panel shows promise as a clinical application to guide PARP inhibitors or platinum drugs used in the treatment of ovarian and other cancers.

## Graphical abstract

The authors developed a non‐exonic single‐nucleotide polymorphism (SNP)‐based targeted next‐generation sequencing panel for homologous recombination deficiency (HRD) detection. All the SNPs had a high minor allele frequency in the Chinese population and non‐exonic location. Comprehensive examination using both clinical ovarian cancer tissues and International Cancer Genome Consortium whole‐genome sequencing datasets *in silico* revealed that the panel performed better than the SNP microarray and would be a better choice for HRD detection in Chinese patients.

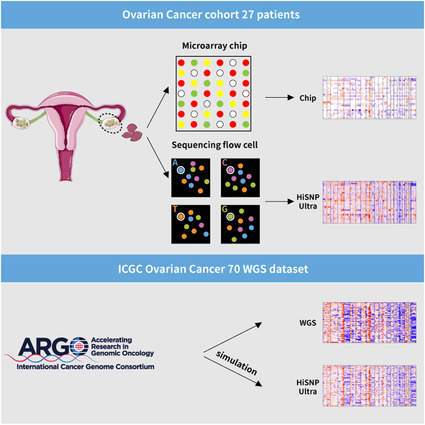

**Abbreviations**
ASCN: allele‐specific copy number
CDx: companion diagnostics
CNV: copy number variant
HRD: homologous recombination deficiency
HRR: homologous recombination repair
ICGC: International Cancer Genome Consortium
LOH: loss of heterozygosity
LST: large‐scale state transitions
MAF: minor allele frequency
NGS: next‐generation sequencing
SNP: single‐nucleotide polymorphism
TAI: telomeric allelic imbalance
Tg‐NGS: targeted next‐generation sequencing
WES: whole‐exome sequencing
WGS: whole‐genome sequencing

## 1 Introduction

Globally, approximately 300 000 new cases of ovarian cancer are diagnosed each year, comprising more than half of all cancer‐related deaths [1, 2]. During the past decade, scientists and physicians have made many efforts to treat ovarian cancer but little improvement in mortality has been observed [3].

A large number of studies have found that about one‐third of ovarian cancers have germline or somatic mutations in homologous recombination genes, which are responsible for homologous recombination repair (HRR) [4]. HRR is one of the core repair methods for DNA double‐strand damage. It uses homologous DNA templates to repair double‐strand breaks. HRR is mainly a DNA repair method to maintain genome integrity and to ensure high‐fidelity inheritance of genetic information [5]. Genomic analysis reveals that more than 50% of high‐grade ovarian serous carcinoma have homologous recombination defects caused by gene mutations or other reasons [6]. The most common mutated HRR‐associated genes in OC are *BRCA1* and *BRCA2*, which together account for 15–25% of OC [7].

The use of PARP inhibitors has shown great promise in clinical studies in patients with *BRCA1/2*‐mutated tumors. In addition to *BRCA*s, ATM, ATR, CHK2 and PALB2 mutations are potential biomarkers for PARP inhibitor sensitivity [8, 9]. Meanwhile, several PARP that have been approved to treat ovarian and breast cancers, also have been proven to be effective in prostate and pancreatic cancer treatments in different clinical settings [10, 11]. Besides genes involved in HR, other germline mutations in genes involved in DNA repair can increase the risk of developing ovarian cancer, and many NGS panels have been designed for detection of mutation in these genes [6, 7, 9, 12, 13, 14, 15, 16].

Several companion diagnostics (CDx) have been approved by the Federal Drug Administration (FDA) for PARPi sensitivity detection; however, defining those patients who may benefit from PARPi therapy remains challenging. Recent studies have shown that analysis of genomic instability as a read‐out for alterations in the HR pathway is a suitable biomarker for a response to PARP inhibition. Homologous recombination deficiency (HRD) causes patterns, such as gene mutations, small or larger fragment deletions/insertions, copy number variants (CNVs) and structural rearrangements, which should be taken into consideration. The main result of HRD is genomic scars, which are comprised of loss of heterozygosity (LOH) [17], telomeric allelic imbalance (TAI) and large‐scale state transitions (LST) [18].

Recently, next‐generation sequencing methods have gradually been replacing single‐nucleotide polymorphism (SNP) microarrays and have become the mainstream method for genomic scar analysis [19]. Considering the tumor specimen ploidy, purity and heterogeneity, to meet the requirements of high coverage and low cost, targeted next‐generation sequencing (Tg‐NGS) anchors the genome‐wide uniform distribution and high heterozygosity rate of tens of 1000s of SNP sites can also apply to the detection of homologous recombination defects. To solve the problems, such as high cost and a massive amount of data for storage and analysis, we developed a new Tg‐NGS and 52 k non‐exonic SNP DNA capture panel, named HiSNP‐Ultra, for genome‐wide CNV and HRD detection. My Choice^®^ HRD CDx and Foundation Focus CDx, which the FDA has approved for genomic scar detection [20], were designed mainly for western populations. The HiSNP‐Ultra panel was developed for the Chinese to make up for the narrow application range of these two panels.

To solve the problem, in this study a new non‐exonic SNP‐based NGS panel available for HRD status detection was designed. We first compared HiSNP‐Ultra Panel with whole‐genome sequencing (WGS) and whole‐exome sequencing (WES) in parallel, with four different analyzing software applications and a series of standards from two independent providers. We then compared it with SNP microarray in CNV and HRD detection on tumor samples of 27 ovarian cancer patients. The HiSNP‐Ultra panel presented an equivalent capacity to the SNP microarray in CNV detection and could be used for HRD status detection to guide clinical platinum‐based or PARP inhibitor treatments, especially for the Chinese population.

## 2 Materials and methods

### 2.1 Standard samples and ovarian cancer sample collection

Standard samples (gDNA) were purchased from Genewell (CA0792‐CA0811, Shenzhen, China) and Cobioer (CGD54143354 etc., Nanjing, China). The study methodologies conformed to the standards set by the Declaration of Helsinki. The experiments were undertaken with the understanding and written consent of each subject. Our study was approved by the Ethics Committee of Henan Cancer Hospital (2021‐KY‐0091‐002). All ovarian cancer samples were collected from Henan Cancer Hospital from 1 January 2014 to 31 January 2022. All patients enrolled were diagnosed with ovarian cancer, which was then detected using a gene chip analysis platform and NGS sequencing technology. Patients who initially responded to platinum‐base chemotherapy, but relapsed 6 or more months after their initial treatment, are classified as ‘platinum‐sensitive’, and those who relapsed within 6 months as ‘platinum‐resistant’.

### 2.2 The design of the HiSNP panel

**Fig. 1:**
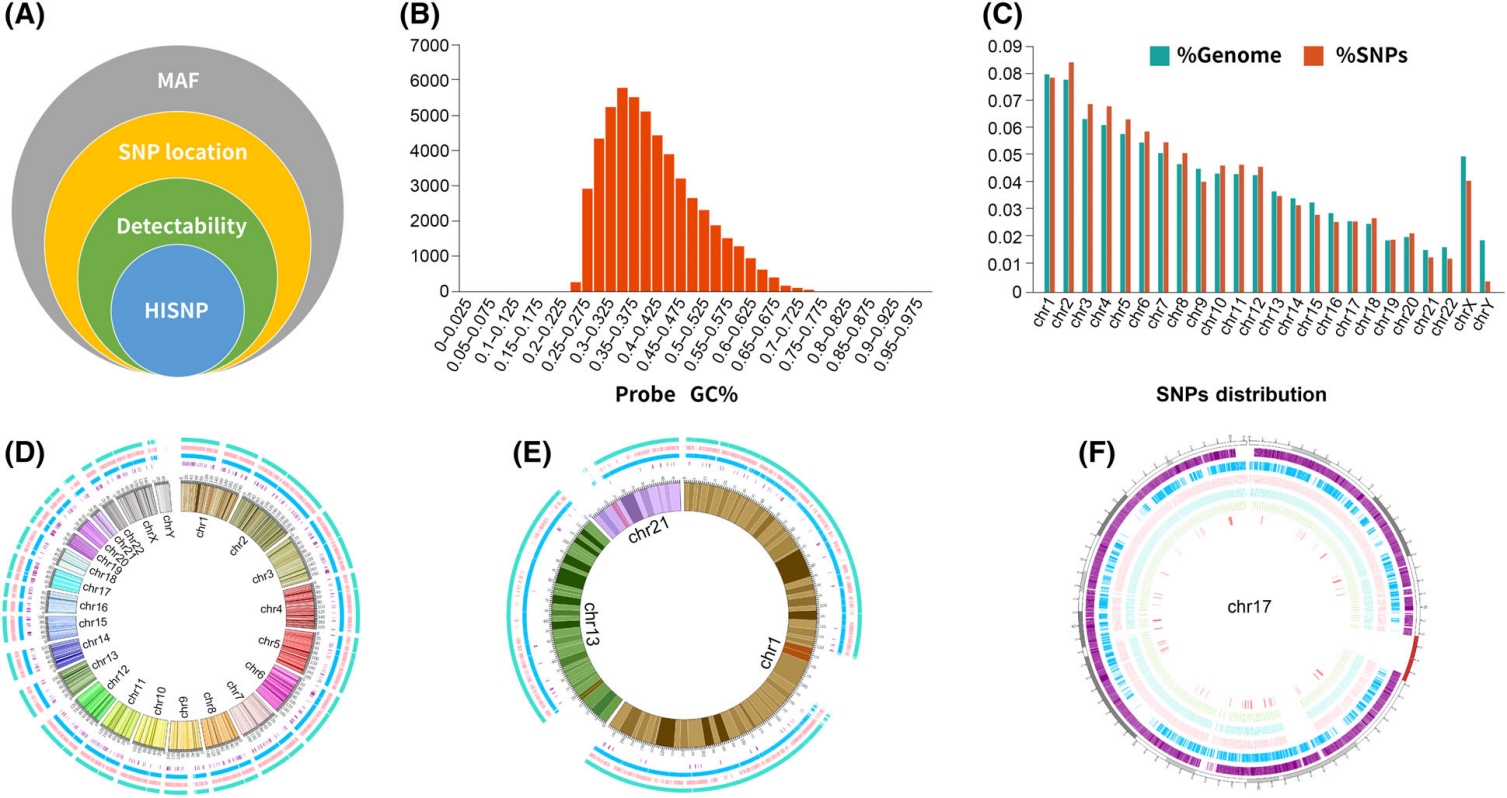
Capture region comparison of four panels. (A) Factors considered in HiSNP panel design. (B) GC% of probes for capturing SNPs in HiSNP panels. GC%, percentage of G and C nucleobases. (C) SNP distribution in each chromosome. (D) The capture region distribution of Affymetrix OncoScan™ Assay (green line), Exome Plus V2 WES panel cover region (orange line), HiSNP‐Ultra panel (blue line) and NanOnco Plus panel v3.0 (purple) with all chromosomes. (E,F) Three randomly selected chromosomes and chromosome 17.

The SNPs targeted by this panel were selected from the 1000 genomes phase 3 variant dataset (https://www.internationalgenome.org/). All 7.5 million SNPs were submitted for custom probe design, with 0.9 million passing the probe design process. From this set of 0.9 million, 52 592 were finally selected. The primary design principles were: (a) the minor allele frequency of SNPs were > 1% in four different races; (b) no exon at 200 bp near SNPs; (c) SNPs with significant deviation from Hardy–Weinberg equilibrium in any of four different races were removed; (d) SNPs cover the genome evenly with an interval of 50 kb; (e) SNPs were selected that had the highest allele frequency in Chinese. The detailed design principles and factors taken into consideration can be found in Tables 1 and S1, Figs. 1 and S1.

**Table 1:** Homologous recombination deficiency detection tools used in this study.

Panel	Cover region size (Mb)	SNP number
HiSNP	1.1	9241
HiSNP‐Plus	3.3	27 225
HiSNP‐Ultra	6.3	52 592
Exome‐V2	46.7	–
OncoScan Chip	–	217 611

### 2.3 DNA extraction

Tumor specimens were collected and paraffin blocks were prepared following the standard process at the Department of Pathology (Henan Cancer Hospital), one slice 4 μm thick was collected and made into an H&E‐stained slide, and about 2–5 pieces 10 μm thick were collected from the paraffin block for DNA extraction. Tissue slices were then continually produced with QIAamp DNA FFPE Tissue Kit (Qiagen, Hilden, Germany) following the manufacturer's instructions, and DNA concentration and purity were quantified by a Qubit fluorescence analyzer (Thermo Fisher Scientific, Waltham, MA, USA), with a minimum concentration of 12 ng·μL^−1^.

### 2.4 Gene chip processing and analysis

Genome‐wide estimation of CNAs was performed using the OncoScan FFPE Assay Kit (Thermo Fisher Scientific) following the manufacturer's instructions. Briefly, 80 ng of FFPE DNA with a volume of 6.6 mL at 12 ng·mL^−1^ was used for the analysis. The DNA was incubated using a PCR amplifier (ABI GeneAmp^®^ PCR system 9700; Thermo Fisher Scientific) with biotin‐labeled Molecular Inversion Probes for 16 h at 58 °C. Subsequently, the circularized DNA is subjected to endonuclease cleavage, which generates fragments 120 bp long and is validated by a capillary electrophoresis apparatus (QIAxecl; Qiagen). The fragments were amplified under the following conditions: 1st PCR 60 °C 30 s/95 °C 1 min/(95 °C 20 s, 60 °C 10 s, 72 °C 10 s)*20 cycles/72 °C 5 min; 2nd PCR 60 °C 30 s/95 °C 1 min/(95 °C 20 s, 60 °C 10 s, 72 °C 10 s)*15 cycles/72 °C 5 min. After amplification, the fragments were subjected to *Hae*III endonuclease digestion and hybridized to OncoScan^®^ CNV FFPE Arrays on Affymetrix Genechip^®^ Hybridization Oven 645 (Thermo Fisher Scientific) set at 49 °C, 0.363 *g* for 16–18 h (overnight), then stained (Affymetrix Genechip^®^ Fluidics Station 450Dx; Thermo Fisher Scientific) and scanned (Affymetrix Genechip^®^ Scanner 3000 7G; Thermo Fisher Scientific) following the manufacturer's instructions. Scanning results from both arrays (AT/GC) were combined and subjected to further analysis in Chromosome Analysis Suite (Thermo Fisher Scientific). As per the manufacturer's recommendation, a gene copy number of <2 was considered a copy number loss, a copy number of >2 was considered a copy number gain, and segments of allelic imbalance were caused by loss of a paternal or maternal allelic gene. After analysis, information on CNAs and LOH was collected.

### 2.5 Next‐generation sequencing library preparation

A DNA librarywas constructed by NadPrep Universal DNA Library Preparation Kit (for MGI) (#1002212; Nanodigmbio, Nanjing, China) from 50 to 100 ng input DNA fragmented with Covaris™ M220 Focused‐ultra sonicator (Covaris, Woburn, MA, USA). Following end‐repair, A‐tailing and adapter ligation, 40 μL of NadPrep SP Beads were used for library clean up and ligated fragments were amplified between 8 and 12 cycles using a 0.5‐m index primers mix. Library Yields were controlled between 500 and 1000 ng. A 60‐μL aliquot of NadPrep SP Beads was used for the recycling library.

### 2.6 Target capture and sequencing

All the standard samples and ovarian cancer sample libraries were single tube capture hybridization. Following the Nanodigmbio Hybridization capture protocol (#1005101; NadPrep Hybrid Capture Reagents, Nanodigmbio), each pool of DNA was combined with 5 μL of 1 mg Cot‐1 DNA (Invitrogen, Carlsbad, CA, USA) and 2 μL each of 0.2 nmol NadPrep NanoBlockers (Nanodigmbio) to prevent cross‐hybridization and minimize off‐target capture. Library and blocker were dried and re‐suspended in hybridization buffer and enhancer. All the standard samples (three GW standards and six KB samples) were captured with the 6.2‐Mb HiSNP‐Ultra panel (Nanodigmbio) and the 46.7‐Mb Exome‐Plus‐V2 WES panel (Nanodigmbio), respectively. In addition to the three GW sample libraries, whole‐genome sequencing was performed with an average depth of 30×. All the ovarian cancer samples were captured with the 8.6‐Mb hybrid capture panel (HiSNP‐Ultra panel and OncoPlus‐V3 panel), which is a 620‐gene panel covering 2.4‐Mbp genome constructed for pan‐cancer (Table S5), and the target capture was performed overnight. Streptavidin M270 (Invitrogen) beads were used to isolate hybridized targets according to the Nanodigmbio Hybridization capture protocol. Captured DNA fragments were amplified with 13–15 cycles of PCR. Libraries were sequenced using 150‐bp paired‐end runs on Illumina platforms (NovaSeq, Illumina, San Diego, CA, USA). All the standard samples and and ovarian cancer sample libraries were sequenced an average of 500×.

### 2.7 HRD score calculation

The sequenced reads were mapped to the reference human genome (GRCh37) using the default parameters in bwa version 0.6.2 (https://github.com/lh3) after removing adaptor and low‐quality reads. Duplicated reads were marked and removed using the MarkDuplicates tool in picard (version 4.0.4.0; Broad Institute, Cambridge, MA, USA). Local realignment around SNVs and indels, as well as quality control assessment, were performed using gatk (version 3.8.0; Broad Institute). Based on gene chip analysis results and next‐generation sequencing mapping data, HRD‐LOH (Loss Of Heterozygosity), HRD‐TA (Telomeric Allelic Imbalance) and HRD‐LST (Large‐Scale Transition) were calculated according to their definition using the r package scarhrd. HRD (Homologous Recombination Deficiency) score was defined as the sum of HRD‐LOH (Loss Of Heterozygosity), HRD‐TAI (Telomeric Allelic Imbalance) and HRD‐LST (Large‐Scale Transition).

- HRD‐LOH (Loss Of Heterozygosity) is defined as segments of LOH with size > 15 Mb but less than a whole chromosome [21].
- HRD‐TAI (Telomeric Allelic Imbalance) is defined as regions of CNA that extend to one of the sub‐telomeres but do not cross the centromere [22].
- HRD‐LST (Large‐Scale Transition) is defined as a chromosomal break between adjacent regions of at least 11 Mb after filtering and smoothing of all variations < 3 Mb (not accounting for the centromeric region) [23].

### 2.8 International Cancer Genome Consortium (ICGC) WGS dataset simulation

‘Ovarian cancer’ was searched in the ICGC database (https://docs.icgc.org/), with the inclusion criteria of WGS and of more than 30× in sequencing depth, and 70 tumor‐normal paired WGS datasets were downloaded. Reads with the same cover regions as these three HiSNP panels (*in silico*) were extracted for the following analysis. sequenza (built‐in scarHRD) was used to analyze the allele‐specific copy number (ASCN) value and HRD score in HiSNP Ultra pane, and titan was used in WGS analysis.

### 2.9 Statistical analysis

Statistical inference was conducted within the r software environment. Statistical significance was set at the 5% level. All *P*‐values and confidence intervals are two‐sided with no adjustment for multiple testing.

Other methods are listed in the Supporting Information.

## 3 Results

### 3.1 The density and uniformity of SNP sites in the three panels

Figure 1A displayed the main factors that we took into consideration in panel design, Fig. 1B showed GC% of probes for capturing SNPs selected in HiSNP panels, and Fig. 1C was the SNP distribution in each chromosome. The distribution of the capture regions of HiSNP‐Ultra Panel with all chromosomes was compared with Affymetrix OncoScan™ Assay, Exome Plus panel (Nanodigmbio) and NanOnco Plus panel v3.0 (Nanodigmbio) (Fig. 1D). Then, chr1, chr13, chr21 together and chr17 were randomly selected to compare the number and spacing of capture regions in the above four panels (Fig. 1E,F). HiSNP‐Ultra panel is designed to detect large‐scale CNVs (at Mb level) to calculate LOH, TAI, LST and HRD scores. Thus the total number of SNP sites could be reduced appropriately. The results showed that the SNP sites of the HRD panel had similar uniformity with Affymetrix OncoScan™ Assay but had fewer SNP sites (52 592 vs. 217 611).

### 3.2 Performance of HiSNP‐Ultra panel on HRD standards

**Fig. 2:**
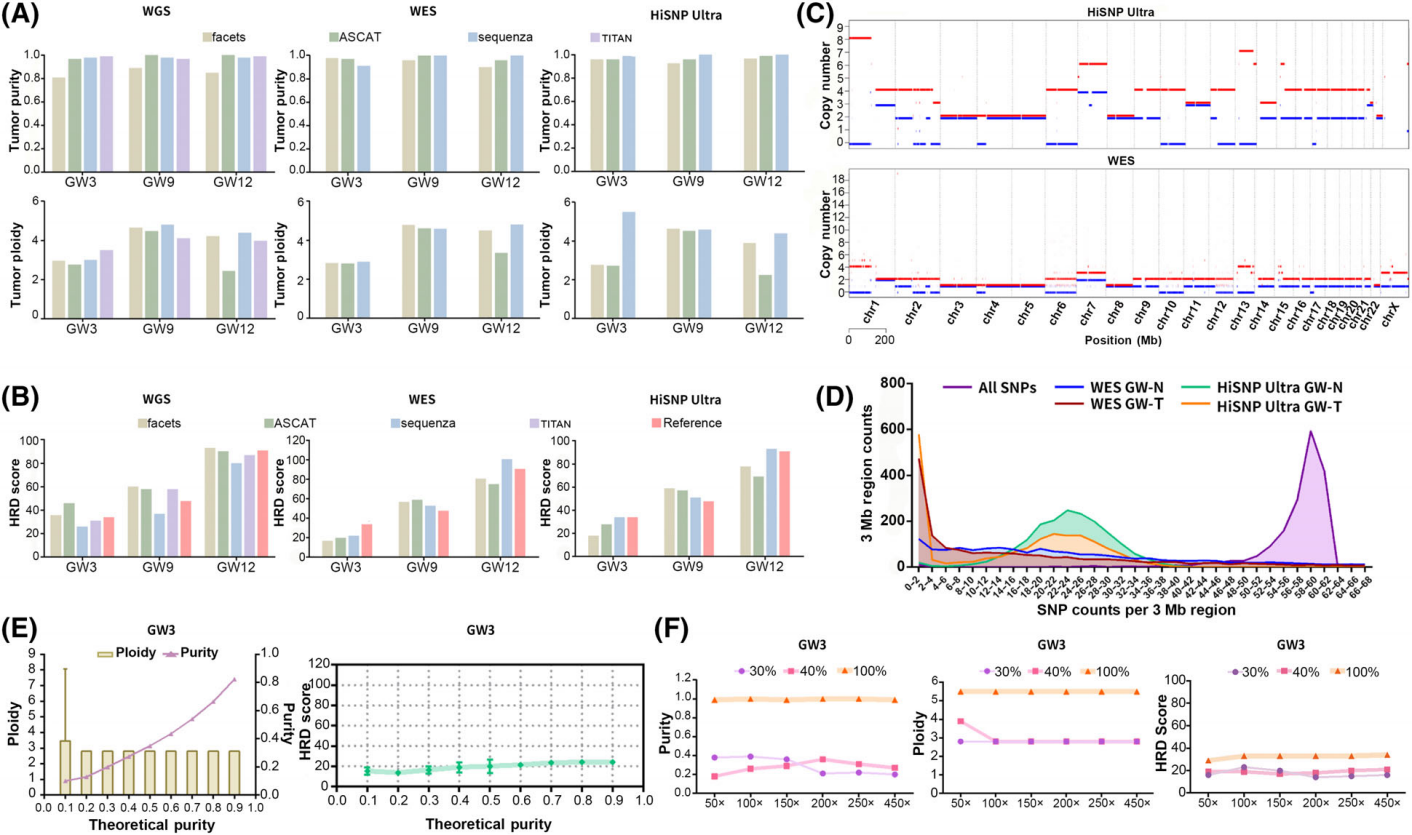
HiSNP Ultra panel assessment based on GW standards. (A,B) Tumor purity, ploidy and HRD score were analyzed as described. (C) The ASCN results detected by WGS and HiSNP‐Ultra Panel were compared. (D) The mean numbers of heterozygous loci within the 3‐m region detected by WES and HiSNP‐Ultra Panel were compared. (E) Tumor purity was simulated *in silico*, and ploidy and HRD scores were calculated; error bar indicates 95% CI. (F) Ploidy and HRD scores were analyzed at different sequencing depths.

With the help of HRD standards (Table S2), we compared the performance of different panels (Fig. 2, Figs S2–S8). The average on‐target rates of the HiSNP‐Ultra panel for all HRD standards were > 88%, and the average sequencing depth was > 300×. The 0.2× mean sequencing depth on some areas of tumor samples was slightly lower than in control samples (~ 96 vs. ~ 98%), suggesting high tumor heterogeneity.

We first performed purity and ploidy analysis using ascat (https://github.com/VanLoo‐lab/ascat), sequenza (https://github.com/cran/sequenza), facets (https://github.com/mskcc/facets) and titan (https://github.com/gavinha/TitanCNA) software, requiring paired tumor and normal tissues for research (Figs 2A and S2). The results showed that the purity detected by different panels was same and the WGS, WES and HiSNP‐Ultra panel results analyzed by different software were different in ploidy analysis. ascat differs significantly from other software in calculating the ploidy of standards with high HRD values. ASCN analysis was performed after the purity and ploidy calculations. From the results, the judgments of the HRD by the HiSNP‐Ultra panel and WGS were consistent with the reference value of standards (Fig. 2B; Fig. S2). For the HiSNP‐Ultra panel, using sequenza (built‐in scarHRD) to analyze the ASCN value was more in line with the reference value. However, HRD scores were closer to the reference value by WGS using the software titan.

To uncover more differences in the detection of CNV and LOH between the HiSNP‐Ultra panel and WGS, we uniformly used scarHRD and the built‐in sequenza software to detect the ASCN of standards (Fig. 2C; Fig. S3). The reproducibility was high (≥ 94%) in the samples of low HRD, and relatively low in the high HRD samples. However, the overall reproducibility was still more than 90% (Figs 2C and S3). To investigate the effect of the homogeneity of the captured region on ASCN and HRD, we divided the whole‐genome into fragments of 3 Mb. The number of heterozygous loci of the standard in each region was counted. The screening conditions were: average sequencing depth > 0.2×, and VAF from 0.3 to 0.7. As shown in Fig. 2D, the number of heterozygous sites captured by the HiSNP‐Ultra panel was mainly about 22–24/3 Mb and the number of WES was 0–4/3 Mb. In addition, due to multiple LOHs in the tumor standard, the number of heterozygous loci in the tumor standard was less than that of the control sample (Fig. 2D). This means that HiSNP‐Ultra can accurately reflect the difference of heterozygous sites between tumor and control samples to improve the detection efficiency of ASCN and LOH than WES.

The detection capabilities of panels and analysis tools are challenged when dealing with samples of low purity. We performed a purity simulation analysis (*in silico*) to verify the relationship between the HRD analysis and the tumor cell purity. The results showed that the purity had a linear relationship with the theoretical percentage and tumor standards’ ploidy and that HRD values tended to be stable when the tumor purity was more than 30% (Figs 2E, S2 and S4). Therefore, we recommend a purity threshold of 30% or above for HiSNP‐Ultra panel detection.

The evaluation of CNV is related to the sequencing depth and software such as sequenza, facets and titan have specific requirements on the sequencing depth for CNV and ASCN analysis. We downsampled the sequencing data for the standards to simulate the impact on HRD analysis using subsets (50×, 100×, 150×, 200×, 250×) of different sequencing depths. In standards with 100% tumor purity, purity and ploidy could be analyzed accurately at ≥ 50× depth (Figs 2F and S5). Considering the variation of tumor purity in the real world, we simulated both 30% and 40% purity to evaluate the impact on HRD analysis. Results revealed that when the sequencing depth was ≥ 200×, the purity data tended to be stable. In addition, HRD itself will lead to the loss of some site information so that the purity calculation results are lower than the theoretical value. Variations of sequencing depths have little effect on ploidy analysis, and the results are stable when depth was ≥ 100×. The HRD analysis results showed that HRD scores at different depths were quite different, and the score tended to level off when the sequencing depth was ≥ 200×. At the same time, the calculation of the HRD score of low HRD samples showed little difference across different depths. However, it was more sensitive to sequencing depth in high HRD samples. Therefore, sufficient sequencing depth is required when using the HiSNP‐Ultra panel to detect actual tumor samples. We recommend a raw sequencing depth of no less than 300×.

**Fig. 3:**
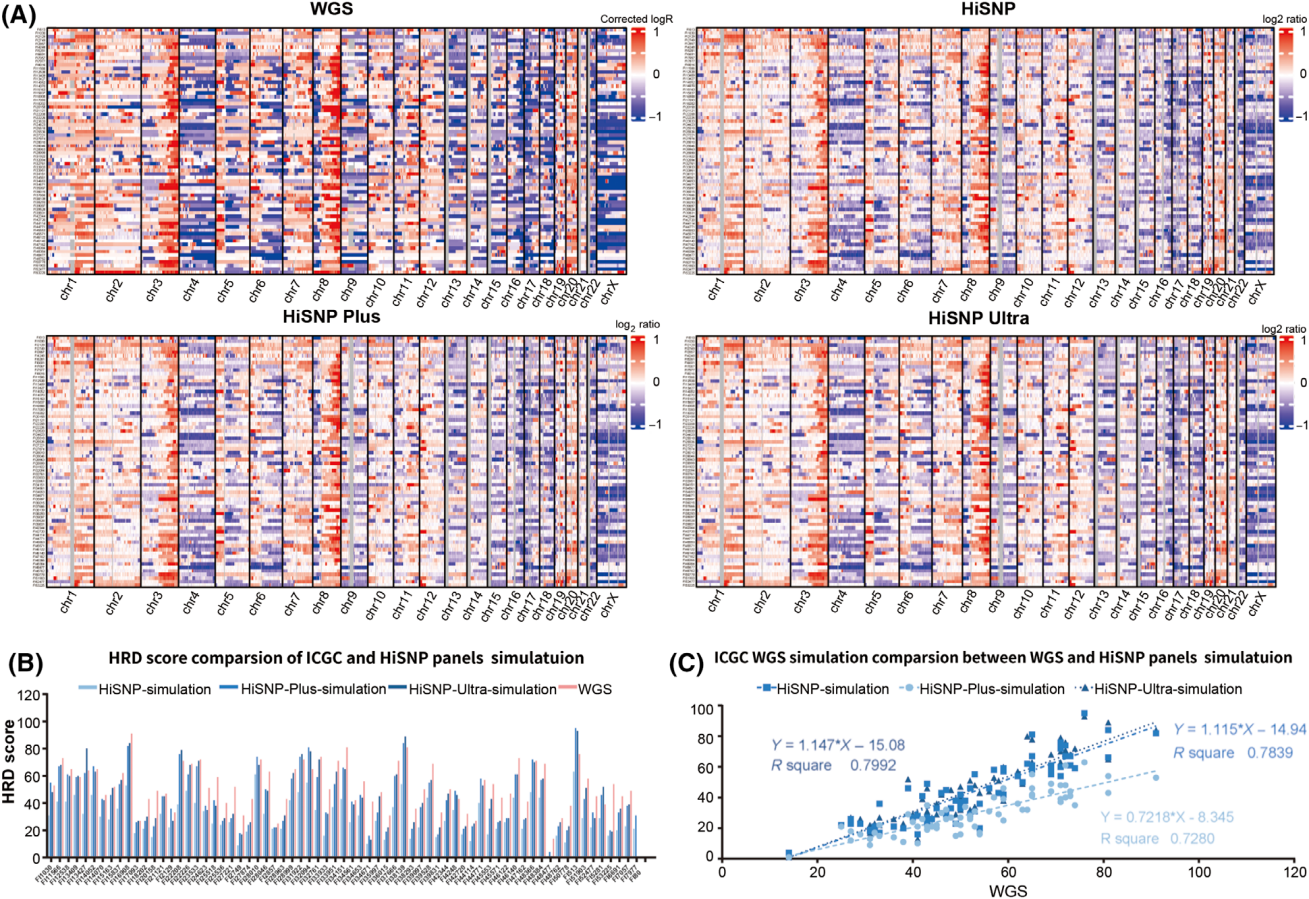
Comparison of HiSNP, HiSNP‐Plus and HiSNP‐Ultra panels with WGS. (A) The whole‐genome CNVs were analyzed by WGS and the three panels (ICGC WGS data of ovarian cancer). (B,C) HRD scores were calculated using the three panels, respectively, and compared with WGS.

Evidence has shown that SNP microarrays still have an advantage over WES for CNV detection. NGS‐target detects minor or novel CNVs that microarrays often miss by providing a base‐by‐base view of the genome. Here, we briefly compare the performance of CNV detection between the HiSNP panels and SNP microarray methods. We found that CNV calls from the HiSNP‐Ultra panel was at least as sensitive as those from microarrays Chip (Fig. 3A). We split the HiSNP‐Ultra panel into another two panels (*in silico*), the HiSNP panel with 9 k SNPs and the HiSNP‐Plus panel with 27k SNPs (Figs S1A and 4B) and then compared them in parallel. The results showed that in the samples with low‐HRD scores, the three panels performed similarly. However, in the samples with medium or high HRD values, the HiSNP‐Plus and HiSNP‐Ultra results were higher than HiSNP and closer to the reference value (Figs S6 and S7).

We also compared HiSNP panels with WGS on CNV and HRD detection to verify them further. All the WGS datasets of ovarian cancer with a sequencing depth of more than 30× were downloaded from the ICGC database; there were 70 valid datasets in total. We extracted SNP sites of the three panels from these WGS datasets and conducted a comparative analysis using WGS as the standard. The comparison results on CNV and HRD are shown separately in Fig. 3A,B. The HRD score cut‐off threshold was defined as 42 [24]; ROC curves of the three panels (*in silico*) were then computed using ICGC WGS data as standard and compared (Fig. S8). The AUC values of HiSNP‐Ultra and HiSNP‐Plus panels are close, a little higher than the HiSNP panel. The results indicated that all three panels had a comparable CNV detection capacity. However, HiSNP‐Ultra and HiSNP‐Plus panels were similar but better than the HiSNP panel in terms of HRD detection.

### 3.3 Performance test of HiSNP‐Ultra panel on clinical ovarian cancer tissues

**Fig. 4:**
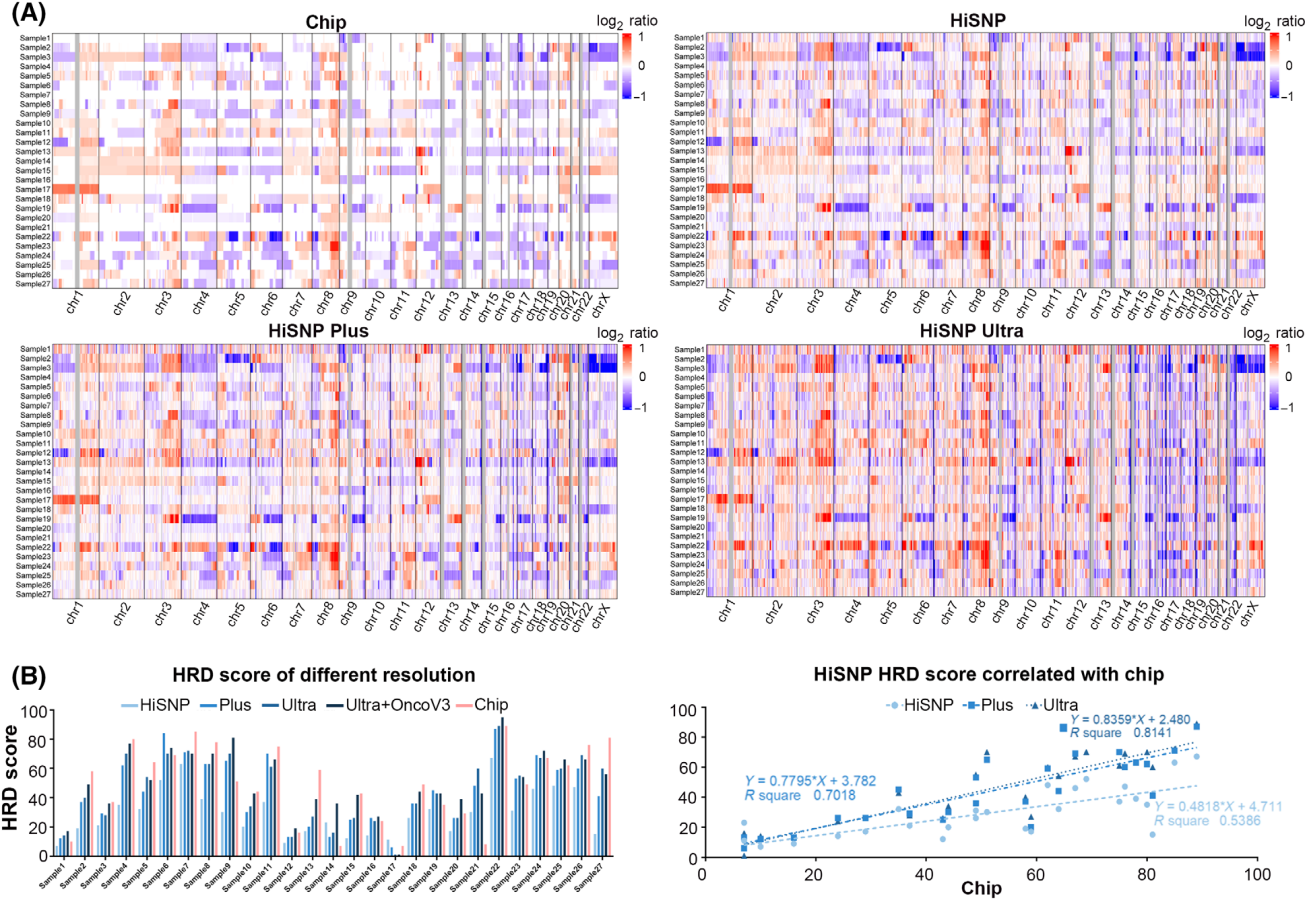
Comparison of HiSNP, HiSNP‐Plus and HiSNP‐Ultra panels with Chip. (A) The whole‐genome CNVs were analyzed by Chip assay and the three panels. (B) HRD scores of OC samples were calculated by the three panels, HiSNP panel, HiSNP‐Plus panel and HiSNP‐Ultra panel, respectively and compared with Chip.

**Fig. 5:**
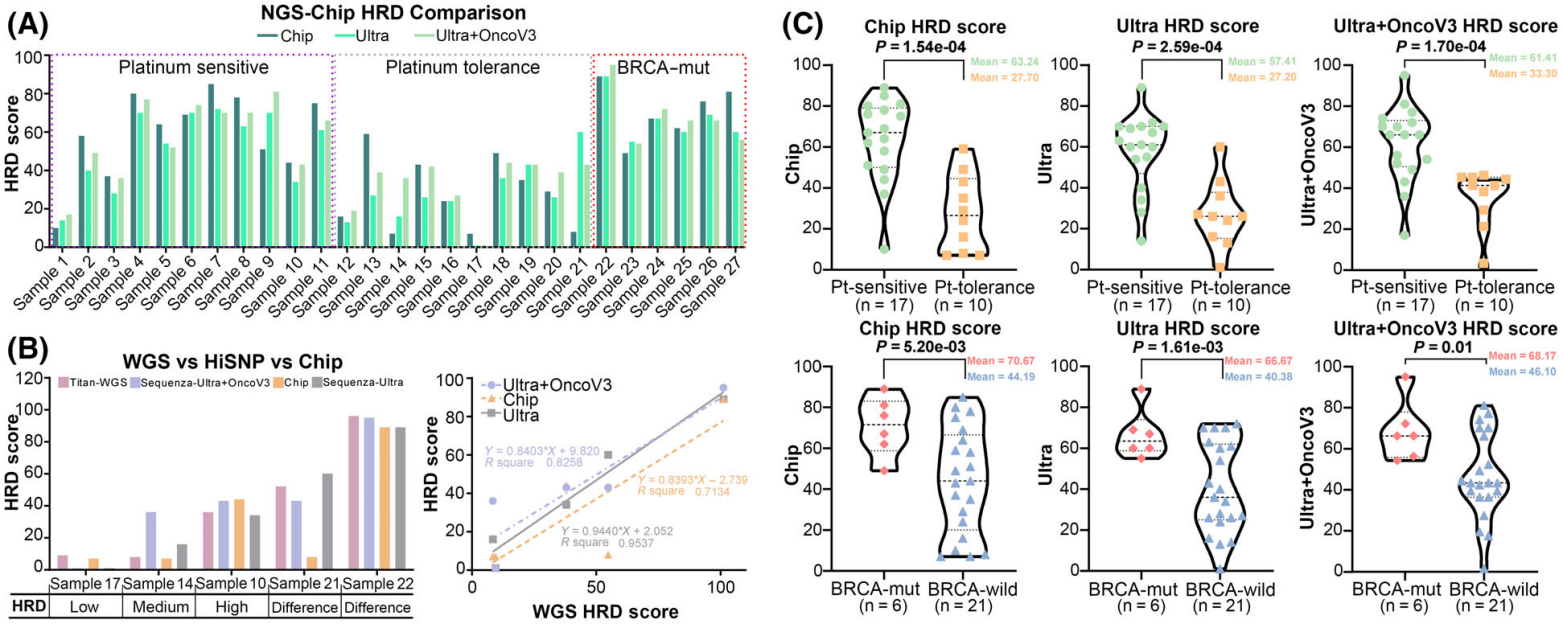
Performance test of HiSNP‐Ultra panel on clinical ovarian cancer tissues. (A) The 27 ovarian tumor samples’ HRD scores based on Chip, Ultra and Ultra+OncoV3 panel. (B) Five ovarian tumor samples’ HRD evaluated by WGS, Chip, Ultra and Ultra+OncoV3 panel. (C) HRD score comparison of two different groups of *BRCA* status and platinum sensitivity. Statistical analysis was performed using an unpaired two‐tailed Student's *t*‐test.

A total of 27 ovarian tumor samples (Table 2) were used to identify the capability of the HiSNP‐Ultra panel in clinical applications. The median scores of LOH, TAI, LST and HRD were 10.5, 23, 14.5 and 43.5, respectively (Table S4). The HRD status is basically consistent with the degree of platinum sensitivity (Fig. 5A). At the same time, we analyzed the driver mutations of these samples sequenced by HiSNP‐Ultra‐NanOnco Plus V3 mixed panel, which targets more than 600 cancer‐related genes including 35 HR genes (Tables S5 and S6). *BRCA1/2* mutations were found in six samples, and scores of LOH, TAI, LST and HRD in the six samples were higher than the cohort median (Table S4). Samples 08, 09 and 10 also have high HRD scores, in which *BRCA1/2* defects were not found; one of the underlying causes may be that HRD genes were downregulated by aberrant methylation of the promoter. Meanwhile, we found that the HiSNP‐Ultra panel has good compatibility with the NanOnco Plus V3 panel (Figs 4B and 5), which means that when HRD and gene mutation analysis are required simultaneously, the combination of HiSNP‐Ultra and exon NGS panel may be a feasible choice.

**Table 2:** Clinical information on the 27 ovarian cancer patients.

No.	Age	Clinical diagnosis	Platinum sensitivity
Sample 1	68	Mucinous adenocarcinoma of right ovary, stage IC2	Sensitive
Sample 2	60	Ovarian carcinosarcoma, stage IIB	Sensitive
Sample 3	60	Ovarian serous carcinoma (high grade), stage IIA	Sensitive
Sample 4	52	Ovarian serous carcinoma stage IIIC	Sensitive
Sample 5	48	Ovarian cancer, poorly differentiated adenocarcinoma, stage III (ct3cn1m0)	Sensitive
Sample 6	42	Ovarian cancer, stage IV (abdominal cavity, liver and spleen metastasis)	Sensitive
Sample 7	31	Bilateral ovarian squamous cell carcinoma, stage III	Sensitive
Sample 8	55	Bilateral ovarian serous carcinoma (high grade), stage IIIC	Sensitive
Sample 9	54	Bilateral ovarian serous carcinoma (high grade), stage IIIC	Sensitive
Sample 10	57	Ovarian serous adenocarcinoma	Sensitive
Sample 11	54	Bilateral ovarian serous carcinoma (high grade), stage IIIB	Sensitive
Sample 12	55	Ovarian serous adenocarcinoma	Tolerance
Sample 13	42	Ovarian serous carcinoma (high grade), stage IIIC	Tolerance
Sample 14	15	Ovarian cancer (mixed germ cell tumor)	Tolerance
Sample 15	55	Ovarian adenocarcinoma, stage IV (supradiaphragmatic, mediastinal lymph nodes, pleural metastasis)	Tolerance
Sample 16	51	Clear cell ovarian cancer	Tolerance
Sample 17	22	Cervical neuroendocrine carcinoma (poorly differentiated), stage IVA (left ovarian metastasis)	Tolerance
Sample 18	45	Ovarian serous carcinoma (high grade), stage IV	Tolerance
Sample 19	59	Bilateral ovarian serous carcinoma (high grade), stage IIIB	Tolerance
Sample 20	56	Ovarian clear cell with serous carcinoma, stage IIIC	Tolerance
Sample 21	42	Ovarian serous carcinoma (high grade), stage IIIC	Tolerance
Sample 22	65	Bilateral ovarian serous carcinoma (high grade), stage IIB	*BRCA*‐mut + Sensitive
Sample 23	50	Bilateral ovarian serous carcinoma (high grade), stage IB	*BRCA*‐mut + Sensitive
Sample 24	48	Bilateral ovarian serous carcinoma, poorly differentiated adenocarcinoma (high grade), stage IIIC	*BRCA*‐mut + Sensitive
Sample 25	44	Bilateral ovarian serous carcinoma, poorly differentiated adenocarcinoma (high grade), stage IV	*BRCA*‐mut + Sensitive
Sample 26	61	Ovarian serous carcinoma, poorly differentiated adenocarcinoma (high grade), stage IIIA1	*BRCA*‐mut + Sensitive
Sample 27	47	Ovarian serous carcinoma, poorly differentiated adenocarcinoma (high grade), stage II	*BRCA*‐mut + Sensitive

To confirm further the performance of the HiSNP‐Ultra panel on HRD detection, we also evaluated the HRD of five OC samples by WGS (Fig. 5B). The results revealed that the HRD score was different when detected by the HiSNP‐Ultra panel and Chip in three samples (Samples 17, 14 and 21), and Chip was more consistent with WGS in the HRD‐low samples. In contrast, the HiSNP‐Ultra panel performed better in the HRD‐high sample. This implies that the HiSNP Ultra panel was superior to Chip for clinical application, since the HRD‐high samples were thought more likely to be sensitive to PARP inhibitors or platinum drugs. We extracted SNPs of the HiSNP and OncoScan from ICGC WGS and our clinical samples WGS datasets and then calculated the heterozygosity rate of these sites in each sample. The results clearly showed the heterozygosity rate of HiSNP is 6% higher than OncoScan in ICGC datasets and 8.7% higher in our clinical datasets (Fig. S9). It indicates that HiSNP is more suitable for HRD detection than OncoScan, especially for the Chinese population.

For 27 ovarian tumor sample sequencing datasets, the results revealed that the HRD score calculated from HiSNP‐Plus and HiSNP‐Ultra were both consistent with Chip (Fig. 4). Sample 21, which was discrepant with WGS, was removed; the *R*‐square values were 0.70 and 0.81, respectively. These results suggest that 27 k SNPs are probably enough to detect the HRD status. According to the clinical information (Table 2), we split the samples into two groups based on *BRCA*‐status or platinum sensitivity. From the HRD score comparison of two different groups (Fig. 5C), the results indicated that both the Chip and HiSNP‐Ultra could distinguish tissues consistent with genetics distinction (*BRCA* status) (Table S5) or clinical distinction (platinum sensitivity) with a *P*‐value < 0.01.

## 4 Discussion

The 2020 edition of the Chinese expert consensus on maintenance therapy for epithelial ovarian cancer recommends that patients with newly diagnosed ovarian cancer should be evaluated for HRD status after pathological diagnosis. Most of the work published to date detecting CNVs is based on an SNP array. This study defined Affymetrix OncoScan™ Assay as one of correlation methods to evaluate the accuracy of HRD panels based on NGS approaches. The density and uniformity of SNP loci are critical for the precision and resolution of CNV identification. Here, we designed an SNP‐based panel, named the HiSNP‐Ultra panel, mainly aimed at the Chinese population. This panel is NGS‐based and has a genome‐wide coverage with a 50 k average interval of each SNP, containing only 52 k SNPs, which is far less than that of Affymetrix OncoScan™ (217 k SNPs). The GC content of captured regions is between 25% and 75%, evenly distributed on chromosomes. These characteristics make it possible to detect HRD accurately with a small amount of data. Another highlight of this panel is that all SNPs are located in the intergenic or intron regions and can be collaboratively used with exon‐targeting panels.

This study performed comparative investigations on the HiSNP‐Ultra panel using HRD standards. We found that HRD analysis results were better than WES. HRD scores vary widely between carcinomas originating from different organs and tissue [21]. In addition, different analysis software has different calculation principles for LOH, LST and TAI and may result in different HRD values. However, our results analyzed by various software were similar, and results by scarHRD are closer to the reference value of the standard.

Allele‐specific copy number analysis is the basis for HRD calculation, which highly relates to tumor sample purity and sequencing depth. Carcinoma tissues with low tumor cell content, such as pancreatic ductal adenocarcinoma, contain large amounts of desmoplastic stroma and may fail to detect HRD accurately [22]. We found that the HiSNP‐Ultra panel was reliable for detecting HRD when the tumor purity was ≥ 30% and the sequencing depth ≥ 200×. The results provide a reference for the clinical application of HRD detection, providing clinicians with evidence on which to formulate individualized treatment plans for patients.

Chip was mostly used for CNV detection, so we compared HiSNP panels with Chip. The results implied that the HiSNP panels were superior to Chip for clinical application because they provided a more accurate prediction in the HRD‐high samples, which were thought to be sensitive to PARP inhibitors or platinum drugs. WGS was considered the most reliable method and even the standard for chromosome variation assessment, because it covers the entire genome, including introns and intergenic regions, and the sequencing depth is relatively even. However, WGS also has some disadvantages, as it is time‐consuming to analyze, has a high cost and requires ample storage space, making clinical application difficult. Therefore, researchers hope to replace WGS with the Tg‐NGS panel for HRD analysis. We compared the HiSNP panels with the WGS method using ICGC WGS datasets. On the basis of the results shown above, we conclude that HiSNP‐Plus (containing 27 k SNPs) was sufficient to detect the HRD status of tumors. Finally, we used 27 OC tissues to investigate panel performance. In five of the tissues, we took WGS as the standard to distinguish the capacities of the panel and Chip for HRD detection. The panel results were more correlated to WGS than was Chip. The HRD scores obtained by panel analysis were more consistent with the platinum sensitivity of clinical practice in the 27 OC cohort, especially in the samples with high or medium sensitivity. This implies that the HiSNP‐Ultra panel was superior to Chip for HRD analysis, at least for OC tissues.

## 5 Conclusions

We have successfully developed an SNP‐based, Tg‐NGS panel for genomic instability detection which has fewer SNPs and is more suitable for Chinese patients than any reported SNP‐based HRD panel. In addition, it can be used cooperatively with regular exon gene panels to detect genomic mutations and CNV comprehensively. This panel is a promising tool for guiding PARPis or platinum‐based therapy in clinical applications. More data is needed to confirm the panel performance. Therefore, a prospective clinical trial, which will recruit more patients of pan‐cancer, has been put on the agenda.

## Conflict of interest

The authors declare no conflict of interest.

## Author contributions

JM, CJ and QX: conceptualization. BW, JZ and CJ: methodology and writing original draft of article. JZ and BW: sample curation. CJ and TC: data analysis and visualization. HZ, MZ, JL, ZW, LD and LW: clinical data and data curation. JM and CJ: project administration. All authors: reviewing and editing the article.

### Peer review

The peer review history for this article is available at https://publons.com/publon/10.1002/1878‐0261.13411.

## Supporting information


**Fig. S1.** Single‐nucleotide polymorphism (SNP) intervals, the ratio of probes with two hits, distribution, minor allele frequency (MAF) and heterozygosity rate of the SNPs in the HiSNP panels.
**Fig. S2.** Tumor purity, ploidy and homologous recombination deficiency (HRD) score of KB standards.
**Fig. S3.** Comparison of standards’ copy number variants (CNVs) calculated by whole‐genome sequencing (WGS) and HiSNP Ultra panel, respectively.
**Fig. S4.** The ploidy and HRD scores of KB standards.
**Fig. S5.** The alteration of ploidy and HRD scores at different sequencing depths.
**Fig. S6.** The correlations of HRD scores by different methods.
**Fig. S7.** HRD scores of standards by different methods.
**Fig. S8.** The ROC curve of the HiSNP panels.
**Fig. S9.** The heterozygosity rate of SNPs of the HiSNP and OncoScan.
**Table S1.** Panel design principles.
**Table S2.** HRD scores of the standards.
**Table S3.** Basic SNP information in the three HiSNP panels.
**Table S4.** HRD scores of the 27 ovarian cancer tissues (detected by HiSNP Ultra+ NanOnco Plus v3).
**Table S5.** Driver gene status in the 27 ovarian cancer tissues.
**Table S6.** Target regions of NanOnco Plus panel v3.0.

## Data Availability

Microarray data from this study are available at GEO (http://www.ncbi.nlm.nih.gov/geo) and are listed under the accession number: GSE203509. Other NGS data are available at SRA (https://www.ncbi.nlm.nih.gov/sra) and are listed under accession number PRJNA850105.
