# Supplementary material for: Improved detection of homologous recombination deficiency in Chinese patients with ovarian cancer: a novel non‐exonic single‐nucleotide polymorphism‐based next‐generation sequencing panel
Source: Mol Oncol. 2023 Mar 16;17(8):1666–77. doi: 10.1002/1878-0261.13411 (PMC10399708; doi:10.1002/1878-0261.13411)
Supplement: Supplementary file 1 — Fig. S1. Single‐nucleotide polymorphism (SNP) intervals, the ratio of probes with two hits, distribution, minor allele frequency (MAF) and heterozygosity rate of the SNPs in the HiSNP panels. Fig. S2. Tumor purity, ploidy and homologous recombination deficiency (HRD) score of KB standards. Fig. S3. Comparison of standards’ copy number variants (CNVs) calculated by whole‐genome sequencing (WGS) and HiSNP Ultra panel, respectively. Fig. S4. The ploidy and HRD scores of KB standards. Fig. S5. The alteration of ploidy and HRD scores at different sequencing depths. Fig. S6. The correlations of HRD scores by different methods. Fig. S7. HRD scores of standards by different methods. Fig. S8. The ROC curve of the HiSNP panels. Fig. S9. The heterozygosity rate of SNPs of the HiSNP and OncoScan. Table S1. Panel design principles. Table S2. HRD scores of the standards. Table S3. Basic SNP information in the three HiSNP panels. Table S4. HRD scores of the 27 ovarian cancer tissues (detected by HiSNP Ultra+ NanOnco Plus v3). Table S5. Driver gene status in the 27 ovarian cancer tissues. Table S6. Target regions of NanOnco Plus panel v3.0. [file MOL2-17-1666-s001.docx]

**Figure S1**

**
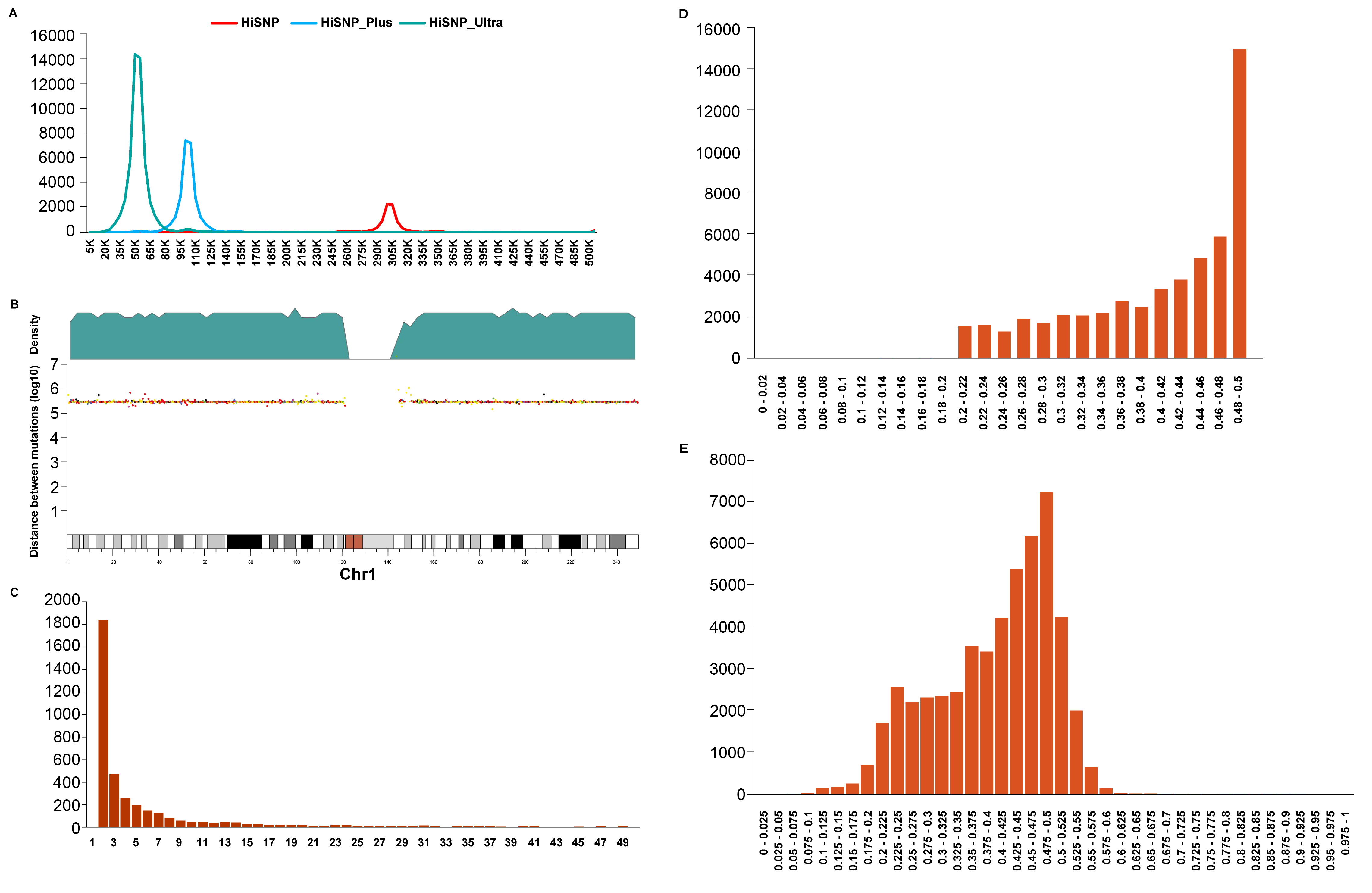
**

SNPs intervals peak for HiSNP、HiSNP-Plus and HiSNP-Ultra Panel(S1A), The ratio of probes with 2 hits or more is 7.24% in HiSNP Panels(S1B), SNPs distribution in each chromosome and chromosome1 in detail(S1C), MAF and Heterozygosis rate of the SNPs in the Chinese population in the database of 1000Genomes(S1D, E).

**Figure S2**


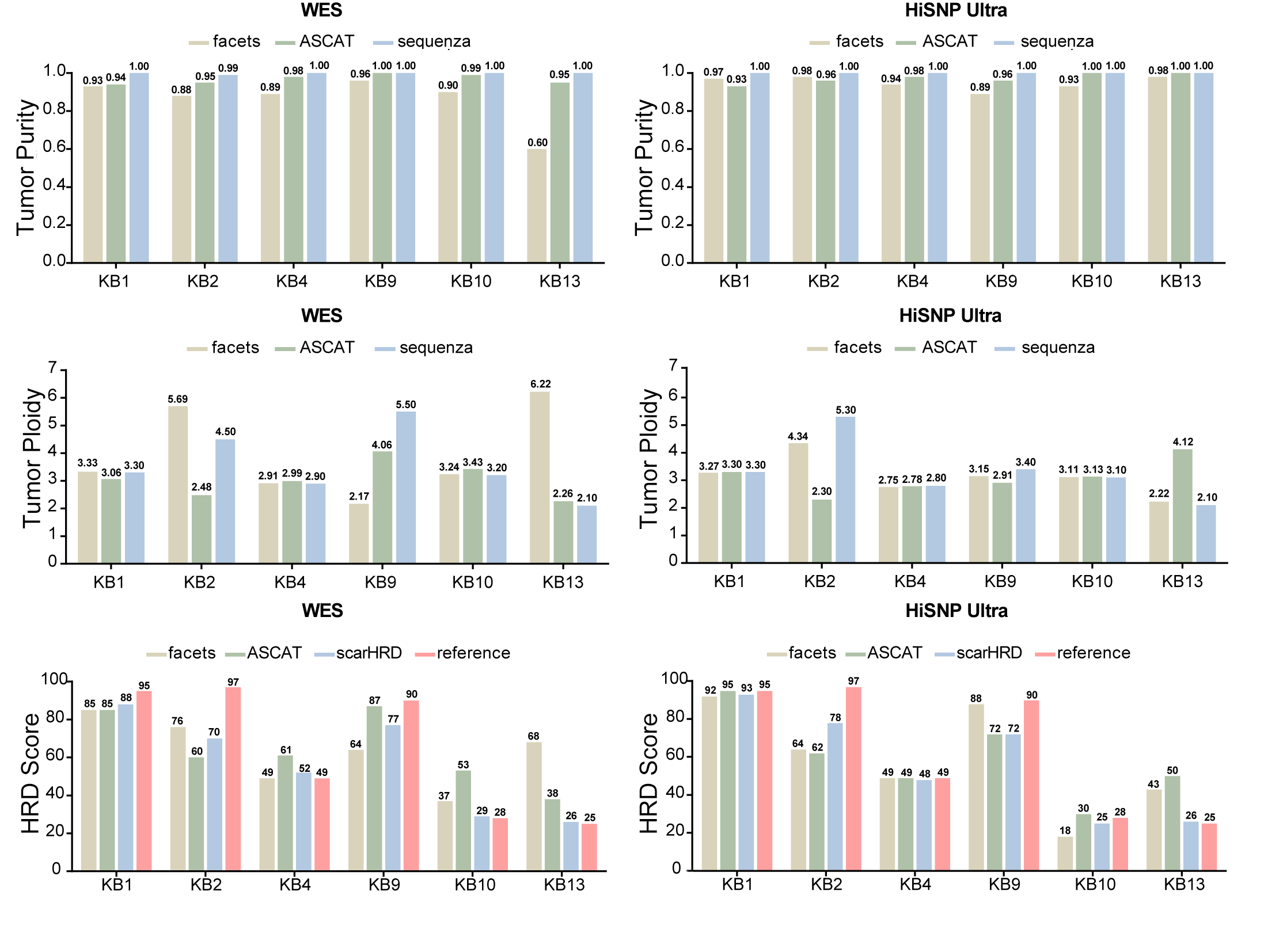


Tumor purity, ploidy, and HRD score of KB standards were analyzed as described.

**Figure S3**


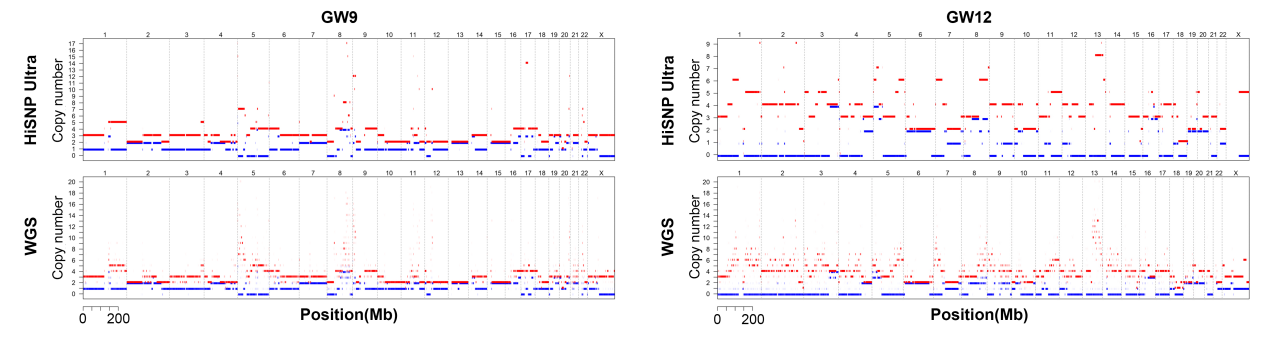


The ASCN results detected by WGS, and HiSNP Ultra Panel were compared using standards.

**Figure S4**


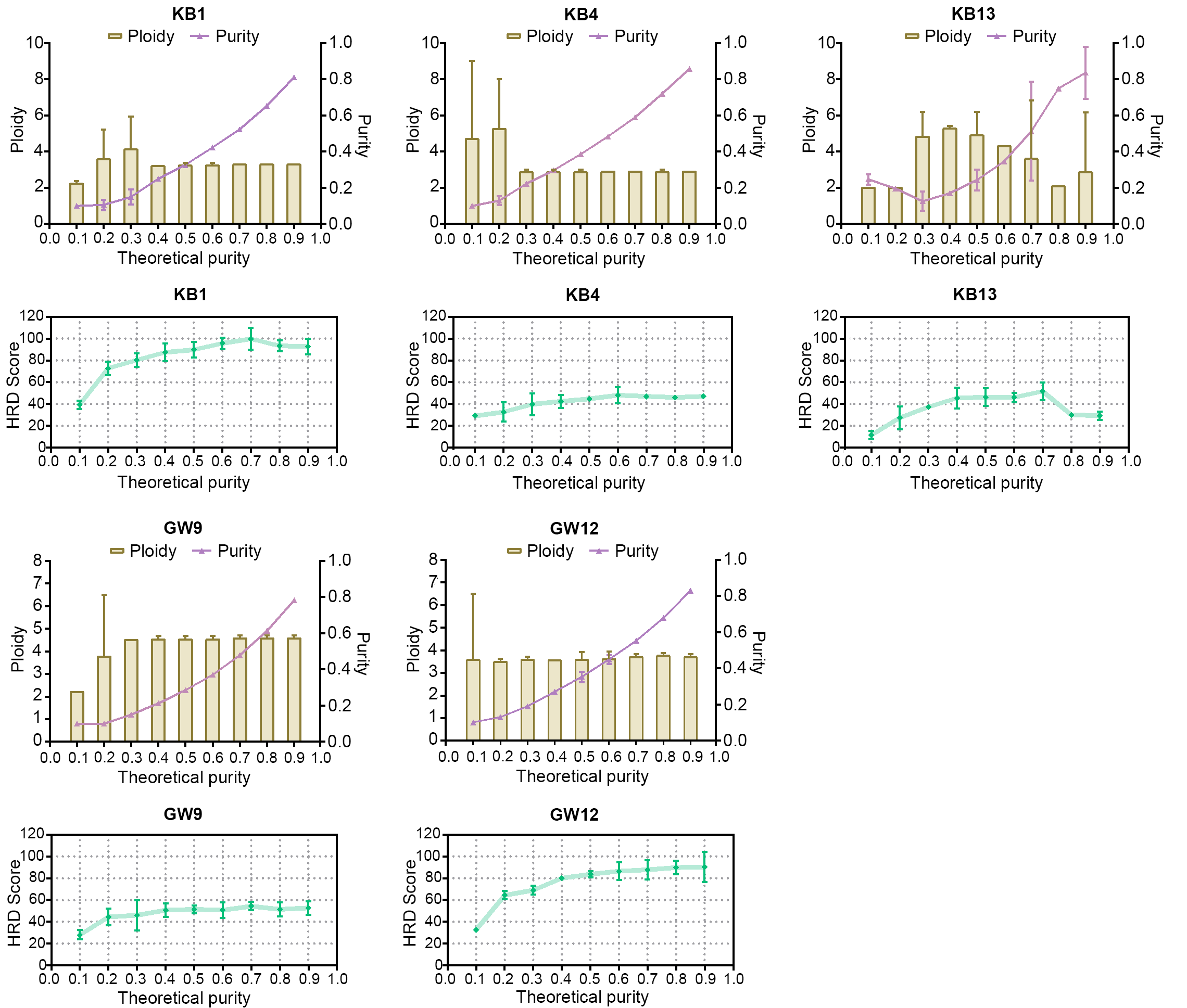


Tumor purity was simulated *in silico*, ploidy and HRD scores of KB and GW standards were calculated.

**Figure S5**


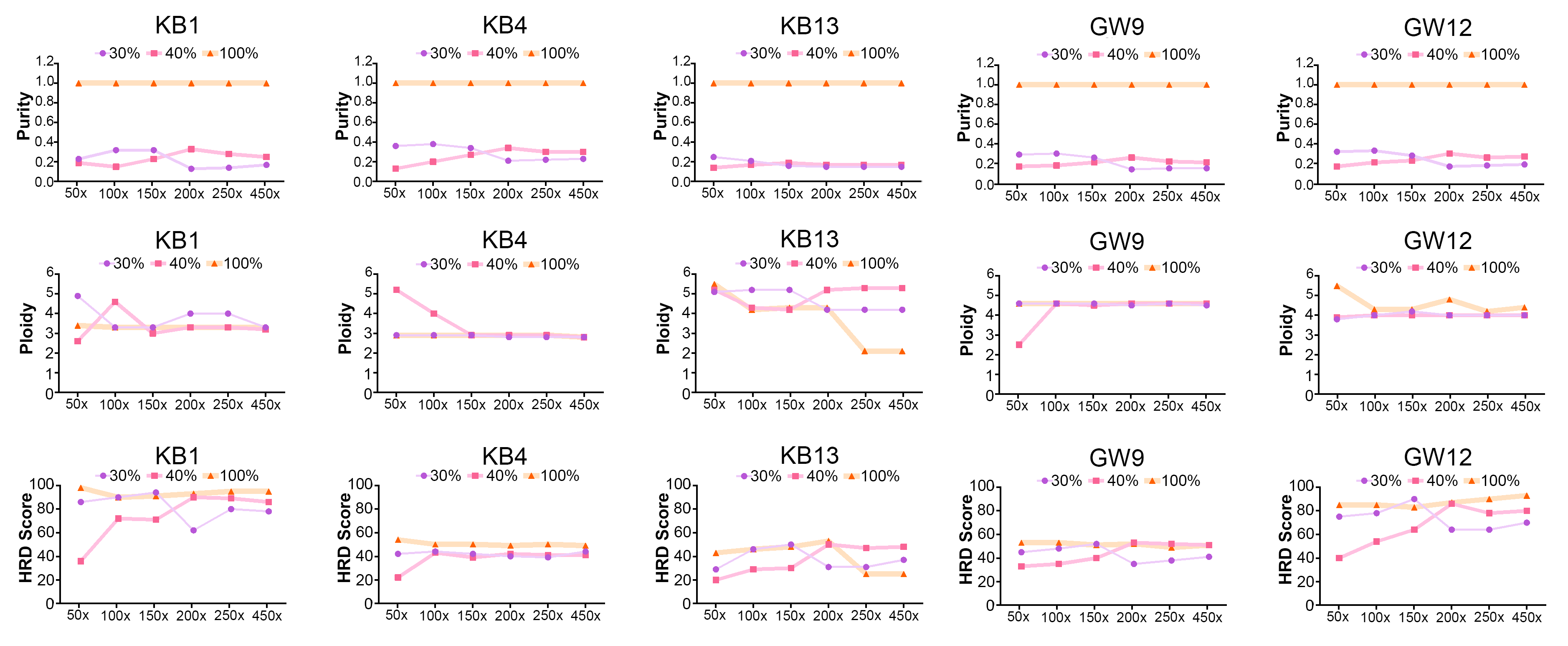


Ploidy and HRD scores of KB standards were analyzed at different sequencing depths.

**Figure S6**

**
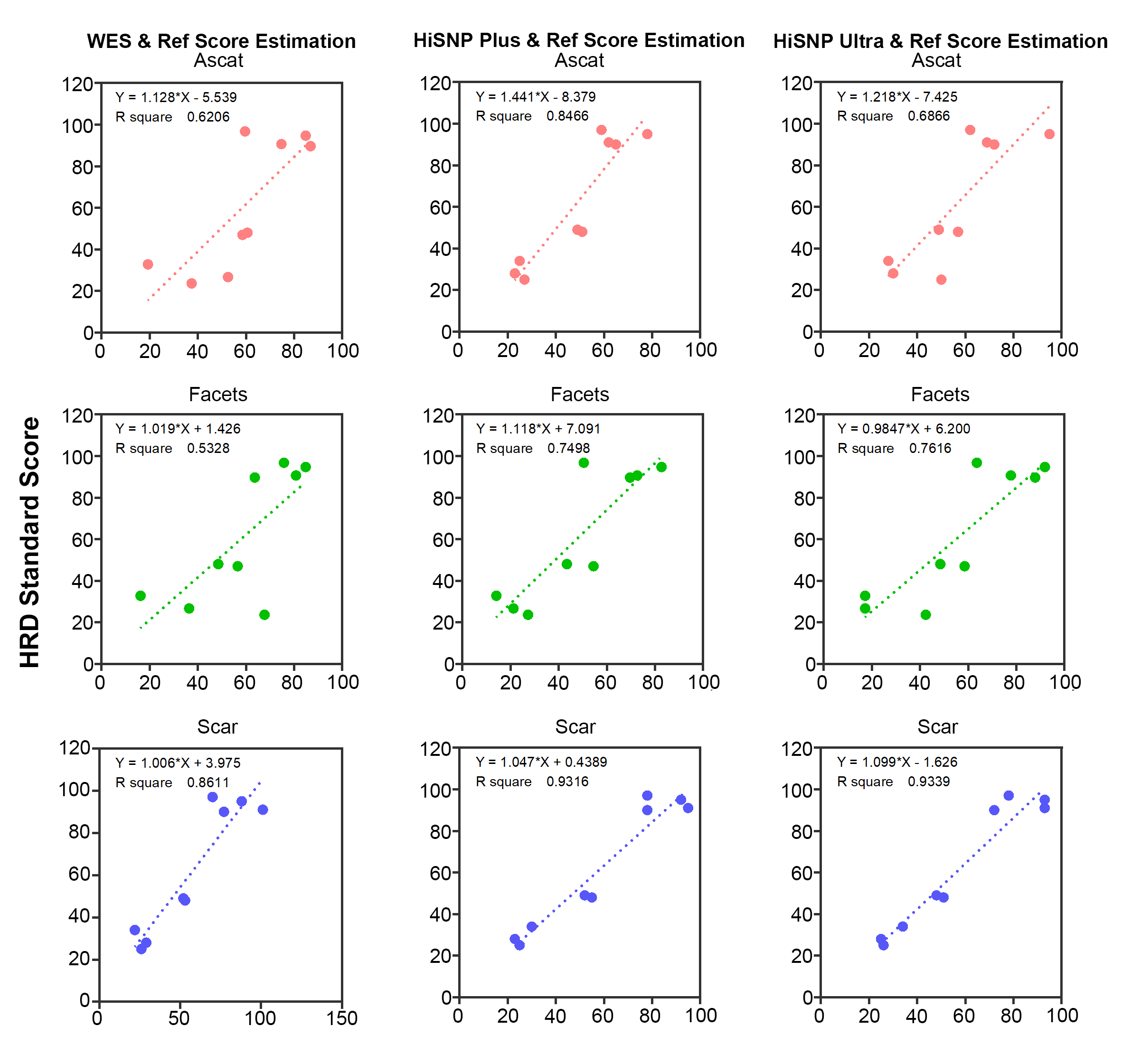
**

The correlations of HRD scores detected by WES, HiSNP Plus, HiSNP Ultra with reference value.

**Figure S7**


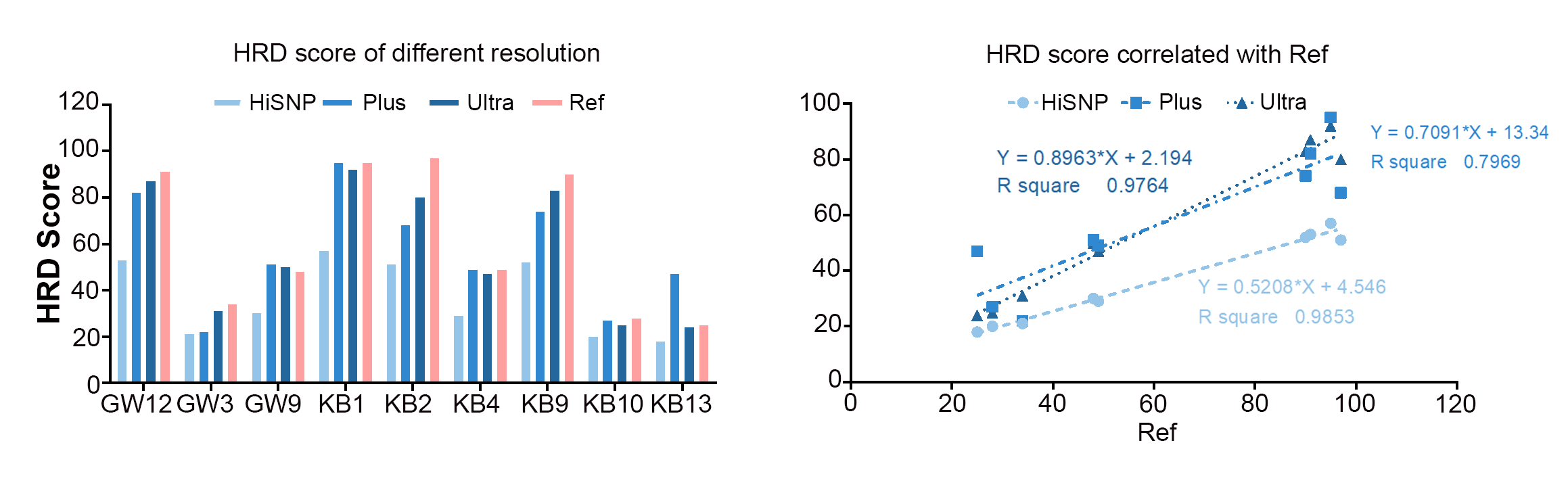
HRD scores of standards were calculated by the three panels respectively and compared with reference.

**Figure S8**

**
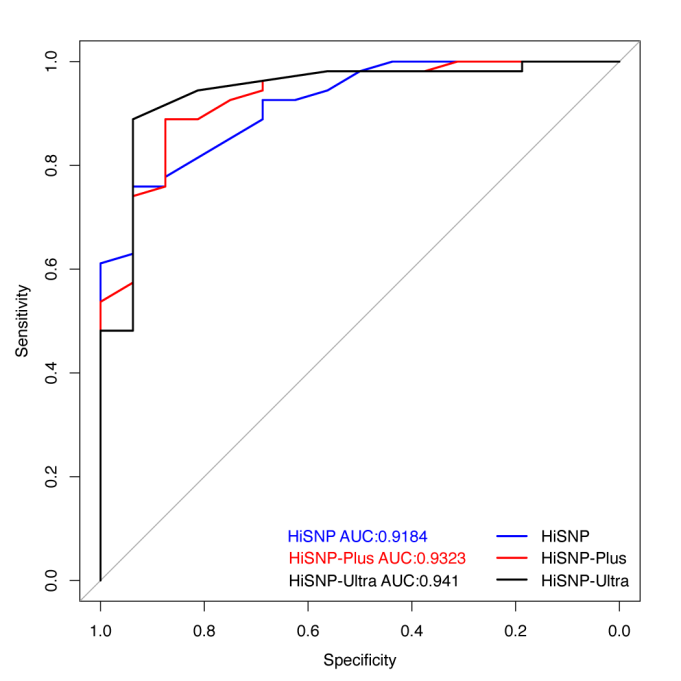
**

The ROC curve of the three panels (*in silico*) using WGS data as standard with a cutoff value (42) for HRD deficiency status

**Figure S9**


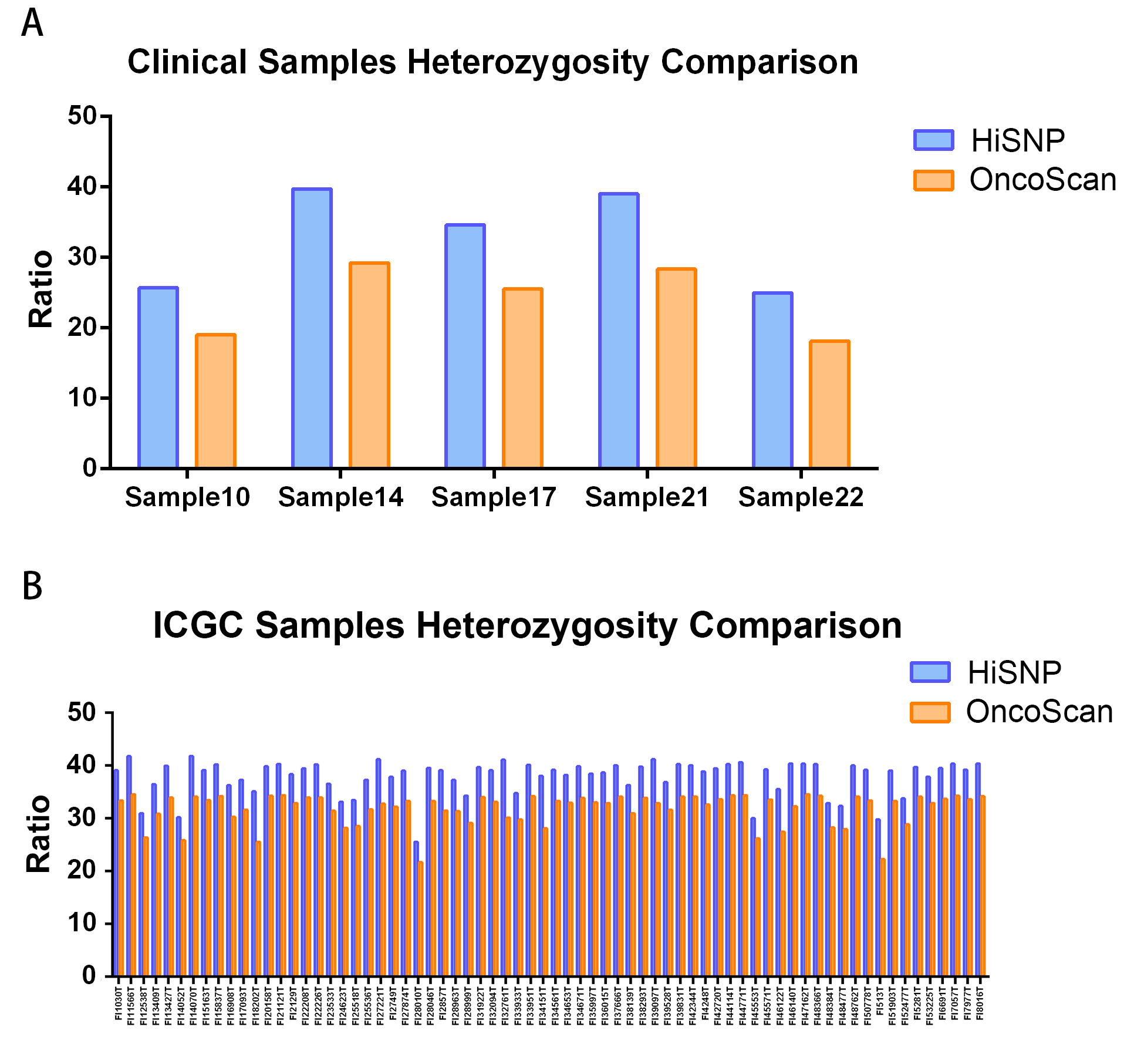


The heterozygosity rate of SNPs of the HiSNP and OncoScan in ICGC and our clinical samples WGS datasets

**Table S1. Panel design principles**

| **Items** | **Parameters** |
| --- | --- |
| Referenced database | 1000 genomes phase 3 variant dataset (<https://www.internationalgenome.org/>) |
| MAF | >1% |
| Location | Intron or intergenic region |
| SNPs interval | 50kb, and no indels within 120bp near |
| GC% of the probes | 25%~75% |
| The percentage of probes with the hit score (<2) | >95% |
| Other considerations | Without Hardy-Weinberg equilibrium or linkage disequilibrium; Prioritize SNPs with high MAF and corresponding high heterozygosity rates in the Chinese population |

**Table S2. HRD scores of the standards**

| **Standards** | **HRD score** | **Standards** | **HRD score** | **Standards** | **HRD score** |
| --- | --- | --- | --- | --- | --- |
| GW3-N | 34 | KB1-N | 95 | KB9-N | 90 |
| GW3-T |  | KB1-T |  | KB9-T |  |
| GW9-N | 48 | KB2-N | 97 | KB10-N | 28 |
| GW9-T |  | KB2-T |  | KB10-T |  |
| GW12-N | 91 | KB4-N | 49 | KB13-N | 25 |
| GW12-T |  | KB4-T |  | KB13-T |  |

**Table S3. Basic SNPs information in the three HiSNP panels**

| **SNPs** | | **AF and the corresponding ratio**  **(1000Genomes)** | | | **MAF and the corresponding ratio**  **(1000Genomes, Chinese population)** | | | **Heterozygosis rate** |
| --- | --- | --- | --- | --- | --- | --- | --- | --- |
| **Density** | **Number** | **0.2-0.8** | **0.25-0.75** | **0.4-0.6** | **0.2-0.5** | **0.25-0.5** | **0.4-0.5** | **≥ 0.4** |
| 300Kb | 9k+ | 99.99% | 85.40% | 34.86% | 99.98% | 92.75% | 62.16% | 57.29% |
| 100Kb | 27k+ | 99.99% | 85.03% | 34.97% | 99.99% | 92.79% | 62.31% | 57.59% |
| 50Kb | 52k+ | 99.99% | 84.89% | 34.70% | 99.99% | 93.00% | 62.68% | 58.02% |

**Table S4. HRD scores of the 27 ovarian cancer tissues (detected by HiSNP Ultra+ NanOnco Plus V3)**

| **NO.** | **LOH** | **TAI** | **LST** | **HRD** |
| --- | --- | --- | --- | --- |
| Sample1 | 4 | 8 | 5 | 17 |
| Sample2 | 10 | 23 | 16 | 49 |
| Sample3 | 7 | 18 | 11 | 36 |
| Sample4 | 21 | 30 | 26 | 77 |
| Sample5 | 10 | 28 | 14 | 52 |
| Sample6 | 21 | 29 | 24 | 74 |
| Sample7 | 25 | 22 | 23 | 70 |
| Sample8 | 18 | 22 | 30 | 70 |
| Sample9 | 10 | 35 | 36 | 81 |
| Sample10 | 12 | 16 | 15 | 43 |
| Sample11 | 21 | 23 | 22 | 66 |
| Sample12 | 4 | 9 | 6 | 19 |
| Sample13 | 10 | 17 | 12 | 39 |
| Sample14 | 5 | 20 | 11 | 36 |
| Sample15 | 11 | 20 | 11 | 42 |
| Sample16 | 6 | 14 | 7 | 27 |
| Sample17 | 0 | 1 | 0 | 1 |
| Sample18 | 9 | 24 | 11 | 44 |
| Sample19 | 12 | 18 | 13 | 43 |
| Sample20 | 11 | 19 | 9 | 39 |
| Sample21 | 4 | 24 | 15 | 43 |
| Sample22 | 27 | 36 | 32 | 95 |
| Sample23 | 12 | 25 | 17 | 54 |
| Sample24 | 21 | 27 | 24 | 72 |
| Sample25 | 16 | 23 | 27 | 66 |
| Sample26 | 19 | 26 | 21 | 66 |
| Sample27 | 12 | 20 | 24 | 56 |
| **Median** | **11** | **22** | **15** | **49** |

**Table S5. Driver gene status in the 27 ovarian cancer tissues**

| **NO.** | **Mutational Genes** | **NO.** | **Mutational Genes** |
| --- | --- | --- | --- |
| Sample1 | *KRAS, TP53, CHEK2* | Sample15 | *TP53* |
| Sample2 | *TP53* | Sample16 | *PIK3CA, CTNNB1* |
| Sample3 | *TP53* | Sample17 | *NA* |
| Sample4 | *NF2, TP53* | Sample18 | *TP53* |
| Sample5 | *TP53* | Sample19 | *TP53* |
| Sample6 | *TP53* | Sample20 | *ARID1A* |
| Sample7 | *TP53* | Sample21 | *NA* |
| Sample8 | *TP53* | Sample22 | *TP53, BRCA1* |
| Sample9 | *TP53,* | Sample23 | *TP53, BRCA1* |
| Sample10 | *TP53* | Sample24 | *TP53, BRCA1* |
| Sample11 | *TP53,* | Sample25 | *TP53, BRCA1* |
| Sample12 | *NA* | Sample26 | *TP53, BRCA1* |
| Sample13 | *TP53* | Sample27 | *BRCA1* |
| Sample14 | *KRAS* |  |  |

**Table S6. Target regions of** **NanOnco Plus Panel v3.0**

| **Full coding sequence** | | | | | | | | | | | | | | | | | | | | | | | | | | |
| --- | --- | --- | --- | --- | --- | --- | --- | --- | --- | --- | --- | --- | --- | --- | --- | --- | --- | --- | --- | --- | --- | --- | --- | --- | --- | --- |
| *ABL1* | | *ABL2* | | | | *ACVR1* | | *ACVR1B* | | | | *AGO2* | | | *AJUBA* | | | *AKT1* | | | | *AKT2* | | | | *AKT3* |
| *ALK* | | *ALOX12B* | | | | *AMER1* | | *ANKRD11* | | | | *APC* | | | *AR* | | | *ARAF* | | | | *ARFRP1* | | | | *ARHGAP35* |
| *ARID1A* | | *ARID1B* | | | | *ARID2* | | *ARID5B* | | | | *ASXL1* | | | *ASXL2* | | | *ATM* | | | | *ATR* | | | | *ATRX* |
| *AURKA* | | *AURKB* | | | | *AXIN1* | | *AXIN2* | | | | *AXL* | | | *B2M* | | | *BABAM1* | | | | *BACH1* | | | | *BAP1* |
| *BARD1* | | *BBC3* | | | | *BCL10* | | *BCL2* | | | | *BCL2L1* | | | *BCL2L11* | | | *BCL2L2* | | | | *BCL6* | | | | *BCOR* |
| *BCORL1* | | *BIRC3* | | | | *BLM* | | *BMPR1A* | | | | *BRAF* | | | *BRCA1* | | | *BRCA2* | | | | *BRD4* | | | | *BRIP1* |
| *BTG1* | | *BTG2* | | | | *BTK* | | *CALR* | | | | *CARD11* | | | *CARM1* | | | *CASP8* | | | | *CBFB* | | | | *CBL* |
| *CCND1* | | *CCND2* | | | | *CCND3* | | *CCNE1* | | | | *CD22* | | | *CD274* | | | *CD276* | | | | *CD70* | | | | *CD74* |
| *CD79A* | | *CD79B* | | | | *CDC42* | | *CDC73* | | | | *CDH1* | | | *CDK12* | | | *CDK4* | | | | *CDK6* | | | | *CDK8* |
| *CDKN1A* | | *CDKN1B* | | | | *CDKN2A* | | *CDKN2B* | | | | *CDKN2C* | | | *CEBPA* | | | *CENPA* | | | | *CFTR* | | | | *CHD2* |
| *CHD4* | | *CHEK1* | | | | *CHEK2* | | *CIC* | | | | *COL4A6* | | | *CREBBP* | | | *CRKL* | | | | *CRLF2* | | | | *CSDE1* |
| *CSF1* | | *CSF1R* | | | | *CSF3R* | | *CTCF* | | | | *CTLA4* | | | *CTNNA1* | | | *CTNNB1* | | | | *CTSO* | | | | *CUL3* |
| *CUL4A* | | *CXCR4* | | | | *CYLD* | | *CYP17A1* | | | | *CYP2D6* | | | *CYSLTR2* | | | *DAXX* | | | | *DCUN1D1* | | | | *DDR1* |
| *DDR2* | | *DDX3X* | | | | *DICER1* | | *DIS3* | | | | *DNAJB1* | | | *DNMT1* | | | *DNMT3A* | | | | *DNMT3B* | | | | *DOT1L* |
| *DROSHA* | | *DUSP4* | | | | *E2F3* | | *EED* | | | | *EGFL7* | | | *EGFR* | | | *EIF1AX* | | | | *EIF4A2* | | | | *EIF4E* |
| *ELF3* | | *EME1* | | | | *EMSY* | | *EP300* | | | | *EPAS1* | | | *EPCAM* | | | *EPHA3* | | | | *EPHA5* | | | | *EPHA7* |
| *EPHB1* | | *EPHB4* | | | | *ERBB2* | | *ERBB3* | | | | *ERBB4* | | | *ERCC1* | | | *ERCC2* | | | | *ERCC3* | | | | *ERCC4* |
| *ERCC5* | | *ERF* | | | | *ERG* | | *ERRFI1* | | | | *ESR1* | | | *ETV1* | | | *ETV6* | | | | *EWSR1* | | | | *EXO1* |
| *EZH1* | | *EZH2* | | | | *EZR* | | *FAAP20* | | | | *FAM175A* | | | *FAM46C* | | | *FAM58A* | | | | *FANCA* | | | | *FANCC* |
| *FANCD2* | | *FANCE* | | | | *FANCF* | | *FANCG* | | | | *FANCI* | | | *FANCL* | | | *FANCM* | | | | *FAS* | | | | *FAT1* |
| *FAT2* | | *FAT4* | | | | *FBXW7* | | *FGF10* | | | | *FGF12* | | | *FGF14* | | | *FGF19* | | | | *FGF23* | | | | *FGF3* |
| *FGF4* | | *FGF6* | | | | *FGFR1* | | *FGFR2* | | | | *FGFR3* | | | *FGFR4* | | | *FH* | | | | *FLCN* | | | | *FLT1* |
| *FLT3* | | *FLT4* | | | | *FOXA1* | | *FOXL2* | | | | *FOXO1* | | | *FOXP1* | | | *FRS2* | | | | *FUBP1* | | | | *FYN* |
| *GABRA6* | | *GALNT12* | | | | *GATA1* | | *GATA2* | | | | *GATA3* | | | *GATA4* | | | *GATA6* | | | | *GEN1* | | | | *GID4* |
| *GLI1* | | *GNA11* | | | | *GNA13* | | *GNAQ* | | | | *GNAS* | | | *GPR124* | | | *GPS2* | | | | *GREM1* | | | | *GRIN2A* |
| *GRM3* | | *GSK3B* | | | | *GTF2I* | | *H3F3A* | | | | *H3F3B* | | | *H3F3C* | | | *HDAC1* | | | | *HDAC2* | | | | *HFM1* |
| *HGF* | | *HIST1H1C* | | | | *HIST1H2BD* | | *HIST1H3A* | | | | *HIST1H3B* | | | *HIST1H3C* | | | *HIST1H3D* | | | | *HIST1H3E* | | | | *HIST1H3F* |
| *HIST1H3G* | | *HIST1H3H* | | | | *HIST1H3I* | | *HIST1H3J* | | | | *HIST2H3C* | | | *HIST2H3D* | | | *HIST3H3* | | | | *HLA-A* | | | | *HLA-B* |
| *HLA-C* | | *HLA-DMA* | | | | *HLA-DMB* | | *HLA-DOA* | | | | *HLA-DOB* | | | *HLA-DPA1* | | | *HLA-DPB1* | | | | *HLA-DQA1* | | | | *HLA-DQA2* |
| *HLA-DQB1* | | *HLA-DQB2* | | | | *HLA-DRA* | | *HLA-DRB1* | | | | *HLA-DRB3* | | | *HLA-DRB4* | | | *HLA-DRB5* | | | | *HMGA2* | | | | *HNF1A* |
| *HOXB13* | | *HRAS* | | | | *HSD3B1* | | *HSP90AA1* | | | | *ICOSLG* | | | *ID3* | | | *IDH1* | | | | *IDH2* | | | | *IFNGR1* |
| *IGF1* | | *IGF1R* | | | | *IGF2* | | *IKBKE* | | | | *IKZF1* | | | *IL10* | | | *IL7R* | | | | *INHA* | | | | *INHBA* |
| *INPP4A* | | *INPP4B* | | | | *INPPL1* | | *INSR* | | | | *IRF2* | | | *IRF4* | | | *IRS1* | | | | *IRS2* | | | | *JAK1* |
| *JAK2* | | *JAK3* | | | | *JUN* | | *KAT6A* | | | | *KBTBD4* | | | *KDM5A* | | | *KDM5C* | | | | *KDM6A* | | | | *KDR* |
| *KEAP1* | | *KEL* | | | | *KIT* | | *KLF4* | | | | *KLHL6* | | | *KMT2A* | | | *KMT2B* | | | | *KMT2C* | | | | *KMT2D* |
| *KNSTRN* | | *KRAS* | | | | *LATS1* | | *LATS2* | | | | *LMO1* | | | *LRP1B* | | | *LTK* | | | | *LYN* | | | | *LZTR1* |
| *MAF* | | *MAGI2* | | | | *MALT1* | | *MAP2K1* | | | | *MAP2K2* | | | *MAP2K4* | | | *MAP3K1* | | | | *MAP3K13* | | | | *MAP3K14* |
| *MAPK1* | | *MAPK3* | | | | *MAPKAP1* | | *MAX* | | | | *MC1R* | | | *MCL1* | | | *MDC1* | | | | *MDM2* | | | | *MDM4* |
| *MECOM* | | *MED12* | | | | *MEF2B* | | *MEN1* | | | | *MERTK* | | | *MET* | | | *MGA* | | | | *MITF* | | | | *MKNK1* |
| *MLH1* | | *MLH3* | | | | *MPL* | | *MRE11* | | | | *MSH2* | | | *MSH3* | | | *MSH6* | | | | *MSI1* | | | | *MSI2* |
| *MST1* | | *MST1R* | | | | *MTAP* | | *MTOR* | | | | *MUTYH* | | | *MYC* | | | *MYCL* | | | | *MYCN* | | | | *MYD88* |
| *MYH9* | | *MYOD1* | | | | *NBN* | | *NCOA3* | | | | *NCOR1* | | | *NEGR1* | | | *NF1* | | | | *NF2* | | | | *NFE2L2* |
| *NFKBIA* | | *NKX2-1* | | | | *NKX3-1* | | *NOTCH1* | | | | *NOTCH2* | | | *NOTCH3* | | | *NOTCH4* | | | | *NPM1* | | | | *NRAS* |
| *NRG1* | | *NRIP1* | | | | *NSD1* | | *NT5C2* | | | | *NTHL1* | | | *NTRK1* | | | *NTRK2* | | | | *NTRK3* | | | | *NUF2* |
| *NUP93* | | *P2RY8* | | | | *PAK1* | | *PAK3* | | | | *PAK7* | | | *PALB2* | | | *PARK2* | | | | *PARP1* | | | | *PARP2* |
| *PARP3* | | *PARP4* | | | | *PAX5* | | *PBRM1* | | | | *PDCD1* | | | *PDCD1LG2* | | | *PDGFB* | | | | *PDGFRA* | | | | *PDGFRB* |
| *PDK1* | | *PDPK1* | | | | *PEG3* | | *PGR* | | | | *PHOX2B* | | | *PIK3C2B* | | | *PIK3C2G* | | | | *PIK3C3* | | | | *PIK3CA* |
| *PIK3CB* | | *PIK3CD* | | | | *PIK3CG* | | *PIK3R1* | | | | *PIK3R2* | | | *PIK3R3* | | | *PIM1* | | | | *PLCG2* | | | | *PLK2* |
| *PMAIP1* | | *PMS1* | | | | *PMS2* | | *PNRC1* | | | | *POLD1* | | | *POLE* | | | *POT1* | | | | *PPARG* | | | | *PPM1D* |
| *PPP2R1A* | | *PPP2R2A* | | | | *PPP4R2* | | *PPP6C* | | | | *PRDM1* | | | *PRDM14* | | | *PREX2* | | | | *PRKAR1A* | | | | *PRKCI* |
| *PRKD1* | | *PRKDC* | | | | *PRSS1* | | *PRSS8* | | | | *PTCH1* | | | *PTEN* | | | *PTP4A1* | | | | *PTPN11* | | | | *PTPRD* |
| *PTPRO* | | *PTPRS* | | | | *PTPRT* | | *QKI* | | | | *RAB35* | | | *RAC1* | | | *RAC2* | | | | *RAD21* | | | | *RAD50* |
| *RAD51* | | *RAD51B* | | | | *RAD51C* | | *RAD51D* | | | | *RAD52* | | | *RAD54B* | | | *RAD54L* | | | | *RAF1* | | | | *RANBP2* |
| *RARA* | | *RASA1* | | | | *RB1* | | *RBBP8* | | | | *RBM10* | | | *RECQL* | | | *RECQL4* | | | | *REL* | | | | *RET* |
| *RFWD2* | | *RHBDF2* | | | | *RHEB* | | *RHOA* | | | | *RIC8A* | | | *RICTOR* | | | *RINT1* | | | | *RIT1* | | | | *RNF43* |
| *ROBO2* | | *ROS1* | | | | *RPA1* | | *RPA3* | | | | *RPS6KA4* | | | *RPS6KB2* | | | *RPTOR* | | | | *RRAGC* | | | | *RRAS* |
| *RRAS2* | | *RSPO2* | | | | *RTEL1* | | *RUNX1* | | | | *RUNX1T1* | | | *RXRA* | | | *RYBP* | | | | *SDHA* | | | | *SDHAF2* |
| *SDHB* | | *SDHC* | | | | *SDHD* | | *SESN1* | | | | *SESN2* | | | *SESN3* | | | *SETD2* | | | | *SETD8* | | | | *SF3B1* |
| *SGK1* | | *SH2B3* | | | | *SH2D1A* | | *SHKBP1* | | | | *SHOC2* | | | *SHQ1* | | | *SIN3A* | | | | *SLFN11* | | | | *SLIT2* |
| *SLX4* | | *SMAD2* | | | | *SMAD3* | | *SMAD4* | | | | *SMARCA4* | | | *SMARCB1* | | | *SMARCD1* | | | | *SMO* | | | | *SMYD3* |
| *SNCAIP* | | *SOCS1* | | | | *SOS1* | | *SOX10* | | | | *SOX17* | | | *SOX2* | | | *SOX9* | | | | *SPEN* | | | | *SPINK1* |
| *SPOP* | | *SPRED1* | | | | *SPTA1* | | *SRC* | | | | *SRSF2* | | | *STAG2* | | | *STAT3* | | | | *STAT4* | | | | *STAT5A* |
| *STAT5B* | | *STK11* | | | | *STK19* | | *STK40* | | | | *SUFU* | | | *SUZ12* | | | *SYK* | | | | *TAF1* | | | | *TAP1* |
| *TAP2* | | *TBX3* | | | | *TCEB1* | | *TCF3* | | | | *TCF7L2* | | | *TEK* | | | *TERC* | | | | *TERT* | | | | *TET1* |
| *TET2* | | *TFE3* | | | | *TGFBR1* | | *TGFBR2* | | | | *TIPARP* | | | *TMEM127* | | | *TMPRSS2* | | | | *TNFAIP3* | | | | *TNFRSF14* |
| *TOP1* | | *TOP2A* | | | | *TP53* | | *TP53BP1* | | | | *TP63* | | | *TRAF2* | | | *TRAF7* | | | | *TSC1* | | | | *TSC2* |
| *TSHR* | | *TSPAN31* | | | | *TTF1* | | *TYRO3* | | | | *U2AF1* | | | *UPF1* | | | *VEGFA* | | | | *VHL* | | | | *VTCN1* |
| *WHSC1* | | *WHSC1L1* | | | | *WISP3* | | *WRN* | | | | *WT1* | | | *WWTR1* | | | *XIAP* | | | | *XPO1* | | | | *XRCC2* |
| *XRCC3* | | *YAP1* | | | | *YES1* | | *ZBTB2* | | | | *ZFHX3* | | | *ZNF217* | | | *ZNF423* | | | | *ZNF703* | | | |  |
| Selected Rearrangements | | | | | | | | | | | | | | | | | | | | | | | | | | |
| *ALK*  intron 18 - 19 | | | *BCL2*  3'UTR | | | | *BCR*  intron 8, 13 - 14 | | | | *BRAF*  intron 7 - 10 | | | | | | *BRCA1*  intron 2, 7 - 8, 12, 16, 19 - 20 | | | | | | *BRCA2*  intron 2 | | | |
| *CD74*  intron 6 - 8 | | | *EGFR*  intron 7, 15, 24 - 27 | | | | *ETV4*  intron 5 - 6 | | | | *ETV5*  intron 6 - 7 | | | | | | *ETV6*  intron 5 - 6 | | | | | | *EWSR1*  *i*ntron 6 - 13 | | | |
| *EZR*  intron 9 - 12 | | | *FGFR1*  intron 1, 5, 17 | | | | *FGFR2*  intron 1, 17 | | | | *FGFR3*  intron 17 | | | | | | *FLI1*  intron 3 - 8 | | | | | | *KIT*  intron 16 | | | |
| *KMT2A*  intron 6 - 11 | | | *MET*  intron 1, 14 | | | | *MSH2*  intron 5 | | | | *MYB*  intron 14 | | | | | | *MYC*  intron 1 | | | | | | *NOTCH2*  intron 26 | | | |
| *NTRK1*  intron 8 - 10 | | | *NTRK2*  intron 12, 15 | | | | *NTRK3*  intron 13 - 14 | | | | *NUTM1*  intron 1 | | | | | | *PDGFB*  intron 1 | | | | | | *PDGFRA*  intron 7, 9, 11 | | | |
| *RAF1*  intron 4 - 9 | | | *RARA*  intron 2 | | | | *RET*  intron 7 - 11 | | | | *ROS1*  intron 31 - 35 | | | | | | *RSPO2*  Upstream, 5'UTR, exon 1 - 2, intron 1 | | | | | | *SDC4*  intron 2 | | | |
| *SLC34A2*  intron 4 | | | *TMPRSS2*  intron 1 - 3 | | | |  | | | |  | | | | | |  | | | | | |  | | | |
| **Microsatellites** | | | | | | | | | | | | | | | | | | | | | | | | | | |
| BAT-25 | BAT-26 | | | | BAT-40 | | | | BAT-RII | | | | NR-21 | | | NR-22 | | | | NR-24 | NR-27 | | | | MONO-27 | |
| D2S123 | D5S346 | | | | D17S261 | | | | D17S520 | | | | D17S250 | | | D18S34 | | | |  |  | | | |  | |
| **Drug response SNPs** | | | | | | | | | | | | | | | | | | | | | | | | | | |
| rs1801133 | | | | rs1801268 | | | | | | rs67376798 | | | | rs1801160 | | | | | rs3918290 | | | | | rs72549303 | | |
| rs1801159 | | | | rs1801158 | | | | | | rs56038477 | | | | rs78060119 | | | | | rs75017182 | | | | | rs72549306 | | |
| rs72549309 | | | | rs1801265 | | | | | | rs396991 | | | | rs4673993 | | | | | rs10929302 | | | | | rs3064744 | | |
| rs1800460 | | | | rs1800462 | | | | | | rs186364861 | | | | rs116855232 | | | | | rs147390019 | | | | | rs45445694 | | |
| rs75467367 | | | | rs74478221 | | | | | | rs766507177 | | | | rs28371735 | | | | | rs1135835 | | | | | rs1135833 | | |
| rs5030867 | | | | rs16947 | | | | | | rs5030656 | | | | rs72549352 | | | | | rs35742686 | | | | | rs3892097 | | |
| rs28371706 | | | | rs28371704 | | | | | | rs28371703 | | | | rs201377835 | | | | | rs1065852 | | | | | rs769258 | | |
| rs59086055 | | | | rs115232898 | | | | | | rs1142345 | | | | rs1051266 | | | | | rs59421388 | | | | | rs1058164 | | |
| rs55886062 | | | | rs2297595 | | | | | | rs1800584 | | | | rs1135840 | | | | | rs28371725 | | | | | rs61736512 | | |
| rs17376848 | | | | rs1801266 | | | | | | rs4148323 | | | | rs25487 | | | | | rs1058172 | | | | | rs5030655 | | |
